# Evaluating the role of Pleistocene refugia, rivers and environmental variation in the diversification of central African duikers (genera *Cephalophus* and *Philantomba*)

**DOI:** 10.1186/s12862-017-1054-4

**Published:** 2017-09-06

**Authors:** Stephan Ntie, Anne R. Davis, Katrin Hils, Patrick Mickala, Henri A. Thomassen, Katy Morgan, Hadrien Vanthomme, Mary K. Gonder, Nicola M. Anthony

**Affiliations:** 1 Department of Biology, Université des Sciences et Techniques de Masuku, B.P.943 Franceville, Gabon; 2 Department of Biological Sciences, University of New Orleans, 2000 Lakeshore Drive, New Orleans, LA 70148 USA; 3 Cheetah Conservation Fund, P.O. Box 1755, Otjiwarongo, Namibia; 4 Comparative Zoology, Institute for Evolution and Ecology, University of Tübingen, Auf der Morgenstelle 28, 72076 Tübingen, Germany; 5 Département Ecologie et Gestion de la Biodiversité, Muséum National d’Histoire Naturelle, CNRS UMR 7179, Avenue du Petit Château, 91800 Brunoy, France; 6 Department of Biology, Drexel University, 3245 Chestnut St., Philadelphia, PA 19104 USA

**Keywords:** Pleistocene, Refugia, Riverine, Barriers, Ecotone, Phylogeography, Generalized dissimilarity models, *Cephalophus*, *Philantomba*, Africa, Ecological gradients

## Abstract

**Background:**

This study aims to assess the role that Pleistocene refugia, rivers and local habitat conditions may have played in the evolutionary diversification of three central African duiker species (*Cephalophus dorsalis, C. callipygus* and *Philantomba monticola*). Genetic data from geo-referenced feces were collected from a wide range of sites across Central Africa. Historical patterns of population genetic structure were assessed using a ~ 650 bp fragment of the mitochondrial control region and contemporary patterns of genetic differentiation were evaluated using 12 polymorphic microsatellite loci.

**Results:**

Mitochondrial analyses revealed that populations of *C. callipygus* and *P. monticola* in the Gulf of Guinea refugium are distinct from other populations in west central Africa. All three species exhibit signatures of past population expansion across much of the study area consistent with a history of postglacial expansion. There was no strong evidence for a riverine barrier effect in any of the three species, suggesting that duikers can readily cross major rivers. Generalized dissimilarity models (GDM) showed that environmental variation explains most of the nuclear genetic differentiation in both *C. callipygus* and *P. monticola*. The forest-savanna transition across central Cameroon and the Plateaux Batéké region in southeastern Gabon show the highest environmentally-associated turnover in genetic variability. A pattern of genetic differentiation was also evident between the coast and forest interior that may reflect differences in precipitation and/or vegetation.

**Conclusions:**

Findings from this study highlight the historical impact of Pleistocene fragmentation and current influence of environmental variation on genetic structure in duikers. Conservation efforts should therefore target areas that harbor as much environmentally-associated genetic variation as possible in order to maximize species’ capacity to adapt to environmental change.

**Electronic supplementary material:**

The online version of this article (10.1186/s12862-017-1054-4) contains supplementary material, which is available to authorized users.

## Background

The tropical forests of central Africa contain some of the most important areas of biological diversity remaining in the world and are important targets for conservation [1–3]. Several hypotheses have been proposed to explain the evolution of such high biodiversity, most of which have either invoked a model of allopatric diversification due to Pleistocene forest refugia and/or riverine barriers or a model of parapatric divergence across ecological gradients [4]. Disentangling these different modes of diversification requires a comprehensive sampling scheme and robust set of analytical tools that can differentiate between competing hypotheses. Mapping patterns of genetic variation across the region can also inform conservation planning by identifying areas where populations may have the best capacity to adapt to environmental change [5]. In this regard, comparative phylogeography and landscape genetics can both make important contributions [6–9].

Of the many tropical biodiversity hypotheses proposed for central Africa, one that has attracted considerable attention is the Pleistocene refugia hypothesis. According to this hypothesis, tropical forests contracted into smaller fragments during the more arid phases of the Pleistocene, leading to the isolation and allopatric diversification of forest-associated taxa [10, 11]. There is considerable support for the role that Pleistocene refugia have played in shaping patterns of biodiversity across equatorial Africa [12–19]. However, there is some debate on the number and location of these refugia within west central Africa [12, 20].

**Fig. 1:**
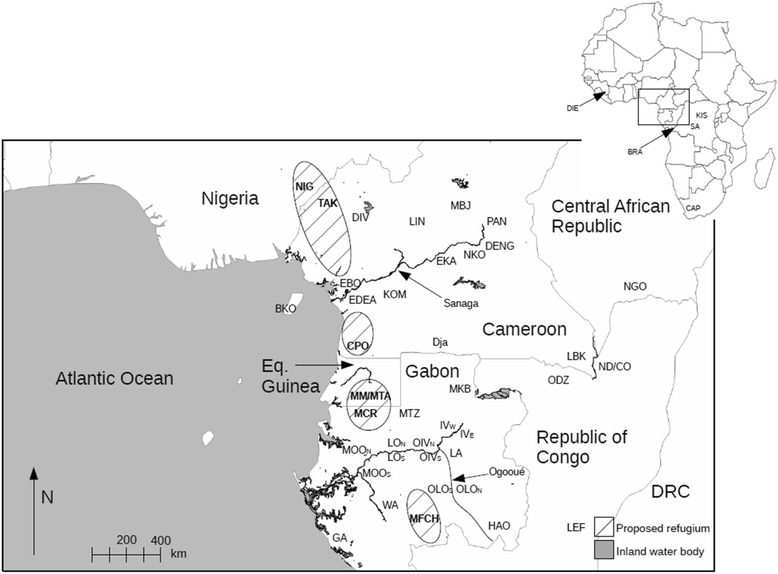
Sites where fecal samples were collected and identified to species level. Arrows indicate the name of the corresponding river. Stippled areas represent hypothesized Pleistocene refugia. Inset to the right shows the location of central Africa in relation to the reset of the continent. Site locations are indicated using a 2–4 digit code which is referenced in Additional file 1: Table S1

While data from contemporary vertebrate and plant species distributions support a single major refugium spanning much of Gabon and Cameroon [21–23], evidence from paleobotanical, palynological and several dispersal-limited plant families (Caesalpinoideae, Rubiaceae and Begoniaceae) indicate that up to four different refugia may have existed [12, 15, 24]. These four refugia are illustrated in Fig. 1 and correspond to the following areas: (a) Gulf of Guinea; (b) south-western Cameroon; (c) Equatorial Guinea and northern Gabon; (d) southern Gabon. Although frequently referred to as lowland in nature [16], candidate refugia in Gabon and Equatorial Guinea are found in hilly areas, whereas the Gulf of Guinea refugium spans the volcanic mountain chain traversing Cameroon and the Gulf of Guinea [25]. Lastly, it has also been proposed that major river basins or smaller gallery forests may have provided additional “fluvial” refugia where more mesic conditions would have allowed forest taxa to persist [12, 26, 27].

In general, researchers recognize three major predictions associated with the Pleistocene refugia hypothesis. Firstly, that fragmentation of formerly contiguous forest into isolated blocks during periods of low rainfall led to the allopatric divergence of forest-associated taxa. Secondly, that forest expansion during moister periods left signatures of population growth in refugial taxa. Lastly, that signals of admixture are to be expected in zones of secondary contact between expanding refugial populations [10, 28, 29]. However, phylogeographic tests of this hypothesis have only been carried out in a handful of taxa and have rarely been addressed in a comparative framework [30–32].

Rivers have also been proposed to act as important drivers of allopatric divergence in tropical systems [33]. Two of the most important predictions of the riverine barrier hypothesis are that sister taxa should be separated by broad rivers and that cross-bank differentiation should increase from headwaters to the mouth [11, 34, 35]. It is also predicted that species with either limited dispersal capabilities or that avoid riparian habitat would show the greatest across-bank differentiation [35–37]. Both the Ogooué and Sanaga rivers appear to be important biogeographic barriers in a number of rodents and primates [19, 38–40]. However, their importance as drivers of diversification in many other taxa is still poorly understood.

Lastly, proponents of the ecological gradient hypothesis have argued that biodiversity patterns formerly attributed to past Pleistocene refugia could have arisen as a result of divergent selection across major ecological gradients [41, 42]. Previous studies in central Africa have shown that the forest-savanna ecotone may represent an important driver of differentiation [43–45]. Similarly, the strong gradient in rainfall between the wetter Atlantic forests of the Gulf of Guinea and the drier semi-evergreen forests of the interior could also be an important factor, as well as the North-South climatic hinge across the equator [30]. Whatever the source of variation, this hypothesis predicts that genetic variation should be structured by environmental heterogeneity rather than by physical or geographic barriers and that genetic differentiation should thus be greatest in areas of sharp ecological transitions between different ecosystems or forest types [46, 47].

Forest duikers (subfamily Cephalophinae) constitute an ideal group for testing mechanisms of evolutionary diversification in African rainforests. Duikers are small to medium sized artiodactyls that are endemic to sub-Saharan region of Africa. Most species are associated with tropical forests and are believed to have originated in the late Miocene [48], making them excellent models for assessing the effect of candidate Pleistocene forest refugia on diversification. Up to six species within this subfamily can occur in sympatry across central Africa [49–52] and their geographic ranges span multiple candidate refugia, rivers, and environmental gradients providing considerable opportunity for assessing competing modes of diversification [53, 54]. Duikers are also amenable to non-invasive genetic sampling and suitable storage and extraction protocols have been developed for this group [55]. Lastly, the three most abundant species in central African rainforests differ in terms of their habitat use, activity patterns, social structure and diet, providing an opportunity to compare phylogeographic patterns in species with contrasting ecologies. While the bay duiker (*Cephalophus dorsalis*) and Peter’s duiker (*C. callipygus*) are restricted to dense forest, the blue duiker (*Philantomba monticola)* occurs in a much wider range of habitats [56] and favors more open areas [57]. *C. dorsalis* and *C. callipygus* are both medium in size and have much larger home ranges than the smaller *P. monticola* [57, 58]. Although all three species are predominantly frugivorous, body size is an important predictor of the size of fruit eaten, thus reducing competition for resources [49, 59]. Species also differ in their activity patterns and degree of social organization. *C. dorsalis* is nocturnal and solitary whereas both *C. callipygus* and *P. monticola* are exclusively diurnal and exhibit different degrees of sociality: *C. callipygus* exhibits a polygynous mating system whereas *P. monticola* is strictly monogamous [57, 60].

The main objective of the present study is to assess the relative roles that candidate Pleistocene refugia, major rivers, and environmental gradients may have played in driving the evolutionary diversification of these three central African forest duiker species. To test these hypotheses, we used mitochondrial sequencing and nuclear microsatellite genotypes obtained from geo-referenced duiker feces. The specific aims of this study are to: (a) determine whether there is a common pattern of allopatric differentiation coincident with the location of hypothesized Pleistocene forest refugia; (b) test for a riverine barrier effect along the Ogooué and Sanaga rivers using a paired cross-bank sampling scheme and (c) examine the effects of geographic distance and environmental variation on genetic structure. We used a suite of methods to examine each of these aims including a generalized dissimilarity modeling (GDM) approach [53] that has the advantage of being able to simultaneously assess the roles of Pleistocene refugia, riverine barriers, geographic distance and environmental heterogeneity on genetic structure and thus discriminate between competing hypotheses of diversification.

## Methods

### Sampling and DNA extraction

A total of 2074 samples (2040 fecal samples and 34 museum and zoo tissues) were collected from 46 sites across nine countries in Africa (Fig. 1; Additional file 1: Table S1). To test for the effect of Pleistocene refugia, fecal samples were obtained from the four major refugia in west Central Africa previously identified by Maley [12]. These were: (1) Gulf of Guinea refugium: Takamanda National Park (TAK) in Cameroon and Cross River National Park (NIG) in Nigeria; (2) South-western Cameroon refugium: Campo Ma’an National Park (CPO) in Cameroon; (3) Equatorial Guinea and northern Gabon refugium: Monte Mitra (MM) and Monte Alén National Park (MTA) in Equatorial Guinea and Monts de Cristal National Park (MCR) in north-western Gabon; (4) Southern Gabon refugium: Birougou National Park (MFCH). Additional sampling was carried out at numerous intervening forest sites across the region, namely: the Gamba complex of protected areas in southern Gabon (GA), Minkébé National Park in north-eastern Gabon (MKB), Nouabelé Ndoki National Park (ND/CO) in the Republic of Congo, Lobéké National Park (LBV) in south-eastern Cameroon, Ngotto forest zone of the Mbaéré-Bodingué National Park (NGO) in south-western Central African Republic, Salonga National Park (SA) in the Democratic Republic of Congo and Bioko island (BKO) in Equatorial Guinea. To explicitly test for an effect of riverine barriers, a cross-bank sampling scheme was used along two putative riverine barriers: the Ogooué (OMA_N_, MOO_N_, MOO_S_, LO_N_, LO_S_, OIV_N_, OIV_S_, LA_N_, OLO_N_, OLO_S_, HAO_N_; where ‘N’ and ‘S’ denote North and South banks of the Ogooué River respectively) and the Sanaga (EDEA, EBO_,_ KOM, EKA, DENG, PAN, LIN, MBJ). Together this sampling strategy encompassed a range of different ecosystem types and environmental gradients, allowing us to also explicitly test the role of environmental heterogeneity in shaping population structure.

At each site, fresh fecal pellets (< 24 h) were sampled opportunistically along a transect line that followed a different compass bearing each day. Replicate samples from the same individual were eliminated on the basis of their multi-locus genotypes (see below). One to four pellets were placed into a vial containing either 5 g of silica gel beads (Sigma, MO) or a vial containing 1.2 ml of RNA*later* (Life Technologies, CA) as previous studies have shown that these two storage methods maximize nuclear and mitochondrial DNA extraction yields [55, 61]. For each fecal sample the following information was collected: site, GPS coordinates and major habitat type in which it was collected. Genomic DNA extraction was carried out using the DNA stool Minikit (Qiagen, CA).

Museum specimens of known geographic origin were also obtained from the following sites, the majority of which could not be sampled otherwise: Diecke, Mount Nimba (DIE) in the Republic of Guinea; Cape province (CAP) in South Africa; Dja Reserve (DJA) and Bamenda (DIV) in Cameroon; Lefini Reserve (LEF), Brazzaville (BRA) and Odzala National Park (ODZ) in the Republic of the Congo; Mbaéré-Bodingué National Park (NGO) in Central African Republic; Mitzic (MTZ) in Gabon; Kisangani (KIS) in the Democratic Republic of the Congo (Additional file 1: Table S1). DNA was either extracted from teeth or from muscle preserved in ethanol as previously described [62]. All fecal and museum extractions were carried out in a dedicated room where no PCR or tissue samples were allowed. Several precautions were taken to minimize the risk of DNA contamination. Extraction blanks and a negative (no DNA) control were included in PCR reactions in order to safeguard against false positives. Fecal DNA extractions and preparation of PCR reactions was carried out in a designated room where only samples from degraded DNA (feces, museum specimens) were allowed. Lastly, only filter tips were used in the preparation of samples for extraction and all work spaces were cleaned with UV light, ethanol and/or bleach prior to PCR amplification.

### Mitochondrial DNA sequencing and microsatellite genotyping

A fragment of the mitochondrial control region of approximately 650 base pairs (bp) in length was amplified and sequenced following previously optimized conditions [62]. Mitochondrial sequences from the three most commonly sampled species (*C. dorsalis, C. callipygus and P. monticola*) were collapsed into representative haplotypes using the program COLLAPSE v.1.2 (available from http://collapse.sharewarejunction.com/). An intraspecific Minimum Spanning Network (MSN) was created with the program NETWORK v.4.6.1.0 [63] in order to visualize relationships between haplotypes.

Fecal samples from these three species were also genotyped at 12 polymorphic microsatellites assembled into three multiplex reactions of four loci each, following previously optimized conditions [61]. A quantitative PCR assay [55] based on a 96 bp of the vertebrate p53 tumor suppressor gene [64] was used to assess nuclear DNA quantity and to determine the number of PCR replicates required to obtain a reliable genotype [65]. In order to minimize the risk of allelic dropout, duiker fecal samples with concentrations of 25–49.9 pg/μl of nuclear DNA or higher were genotyped a minimum of four times and those of 50 pg/μl of DNA or more were typed a minimum of three times [55]. Samples with concentrations <25 pg/μl were not genotyped because of the risk of allelic dropout. The program ARLEQUIN v.3.5.1.3 [66] was used to calculate expected heterozygosity, and deviations from Hardy Weinberg Equilibrium (HWE) and Linkage Equilibrium (LE) within each population. The significance of HWE and LE tests was assessed using an extension of Fisher’s exact probability test with contingency tables [67] and a test analogous to Fishers’s exact test extended to a triangular contingency table of arbitrary size, respectively [68]. GIMLET v.1.3.3 [69] was used to assess the minimum number of typed loci required to differentiate first order relatives (P_ID_ ≤ 0.05). KINGROUP v.2 [70] was used to identify identical genotypes and first order relative relationships between individuals sampled within the same population. Samples with identical genotypes or sharing first order relatives within each population were removed from downstream analyses.

### Species identification

Fecal samples were identified to species level using an established mitochondrial control region diagnostic [62]. We also carried out a phylogenetic analysis to assess whether the highly divergent mitochondrial sequences obtained from samples collected in the Nigerian highlands were from a new species of dwarf duiker *P. walteri* described by Colyn and colleagues [71]. To test this hypothesis, a 402 bp fragment of the mitochondrial cytochrome c oxidase subunit I (COI) gene was amplified from fecal samples using published primers [71]. These sequences were then aligned with COI sequences from *P. maxwelli* (GenBank HQ644099–644100, HM144021, HM 144027), *P. monticola* (GenBank GQ144522–144545, HM144015, HM144016, HM144020, HM144022, HM144024, HM144026), *P. walteri* (GenBank HM144017–144019) and an outgroup species *C. callipygus* using the program MEGA7 [72]. A neighbor-joining tree was then inferred using the Tamura-Nei distance [73] and a bootstrap consensus trees was constructed from 1000 replicates.

Although the majority of fecal samples could be identified on the basis of their mitochondrial DNA, fecal samples from NGO could not be sequenced due to their poor quality and were instead identified on the basis of their multi-locus microsatellite genotypes using a cluster-based analysis implemented in the Bayesian program STRUCTURE v.2.3.3 [74]. To do this, multi-locus microsatellite genotypes were first obtained from reference samples of all six duiker species known to occur in sympatry in the study area (*P. monticola, C. callipygus, C. dorsalis, C. silvicultor, C. leucogaster* and *C. nigrifons*). The identification of each NGO sample was then inferred by observing the group in which it clustered with an estimated membership coefficient (Q) of ≥ 0.9. In these cluster analyses, the total number of steps in the Markov Chain Monte Carlo (MCMC) and burn-in were set to 100,000 and 10,000 generations respectively, with 10 separate simulations for each value of K. The admixture model was used and allele frequencies among populations were assumed to be correlated. STRUCTURE HARVESTER was used to determine the most probable value of K using the ΔK statistics procedure [75]. CLUMPP v.1.1 [76] was used to align all multiple runs for each K (because of label switching) and DISTRUCT v.1.1 [77] was used to make graphical representations of STRUCTURE outputs.

### Pleistocene refugia hypothesis

All mitochondrial DNA analyses only considered sites where three or more individuals had been sampled. In cases where sampling was relatively limited*,* sequences from the following nearby sites (< 100 km) were pooled: (1) TAK, NIG and BAM in the Gulf of Guinea refugium; (2) MTA and MM within the Equatorial Guinea and northern Gabon refugium; (3) ND and CO within the Parc National de Nouabelé Ndoki. A Spatial Analysis of Molecular Variance (SAMOVA) of each species was carried out using the program SAMOVA v.1.0 [78] where the number of groups (K) was varied from 2 to 5. The program ARLEQUIN was also used to conduct a series of Analyses of Molecular Variance (AMOVA) in order to test the role that putative refugia have played in shaping regional genetic structure. Samples were grouped by each of the four putative refugia, namely: Gulf of Guinea (TAK, NIG, BAM), south-western Cameroon (CPO), Equatorial Guinea and northern Gabon (MM, MTA, MCR) and southern Gabon (MCR). All remaining sites were then pooled into the final group. In the case of *P. monticola*, AMOVA analyses were conducted without sequences from *P. walteri* since mitochondrial analyses showed them to be highly divergent from *P. monticola* (see below). The effect of a major fluvial refugium within the Sanaga River basin was assessed by pooling all sites within the river basin into one group and placing all remaining sites into a second group.

Three measures of mitochondrial diversity were calculated for each sampling site using the program DnaSP v.5.1 [79] namely: haplotype diversity (Hd), the average number of nucleotide differences per site between sequences (π) [80] and the mean number of segregating sites per nucleotide (θ) [81]. A Mann-Whitney-Wilcoxon test was used to test the hypothesis that molecular diversity was higher in candidate refugial sites than in intervening sites. Evidence for past demographic expansion within regional groups identified by SAMOVA analyses was assessed using Fu’s Fs and Tajima’s D, as implemented in the program ARLEQUIN.

Patterns of nuclear genetic structure were investigated using STRUCTURE. The total number of steps in the Markov Chain Monte Carlo (MCMC) and burn-in were set respectively to one million and 100,000 generations with 10 separate simulations for each value of K. The admixture model was used and allele frequencies among populations were assumed to be correlated. Clustering of K populations within species was used to assess whether population differentiation was structured by putative refugia. As described above, STRUCTURE HARVESTER was used to determine the most probable value of K using the ΔK statistics procedure, CLUMPP v.1.1 was used to align all multiple runs for each K and DISTRUCT 1.1 was used to make graphical representations of STRUCTURE outputs.

### Riverine barrier hypothesis

An AMOVA analysis of mitochondrial DNA sequence datasets was also carried out to test the effects of the Ogooué and Sanaga rivers on genetic structure of all three species. To do this, sites immediately flanking the north and south banks of each river were placed into two separate groups. To test the combined effects of both Sanaga and Ogooué River bodies, the entire dataset was divided into three groups made up of: (a) sites to the north of the Sanaga; (b) between both rivers and (c) to the south of the Ogooué. In the case of *C. callipygus*, we could not test the effect of the Sanaga River barrier due to insufficient sample sizes. STRUCTURE analyses of nuclear genetic data were also carried out to examine whether genetic variation was partitioned by either river.

### Ecological gradient hypothesis

A generalized dissimilarity modeling (GDM) [82] approach was adopted to test the hypothesis that environmental heterogeneity has played a role in driving genetic differentiation, and to further evaluate the relative roles of Pleistocene refugia and riverine barriers in a single analysis. GDM is an iterative matrix regression method that fits dissimilarities of predictor variables to dissimilarities of a response variable. It can analyze and predict spatial patterns of beta diversity across large areas, by using I-spline basis functions to adjust non-linear relationships between environmental variables and biological variation. GDM was originally designed to predict turnover in community composition across a landscape but has subsequently been modified [5] to predict patterns of intraspecific genetic differentiation as a function of environmental variation, landscape features and geographic distance. Once the relationship between predictor and response variables is known, the spatial distribution of the response variable can be projected across the study area based on spatial variation in the predictor variables.

In the present study, GDM was used to model the relationship between several predictor variables (straight-line geographic distance, environmental variables - Additional file 1: Table S2 -, riverine barriers, and Pleistocene refugia) to a single response variable (pairwise F_ST_ values). To do so, we took a two-step modeling approach, following an established protocol [5]. The first step avoids projecting genetic variation into areas where the target species does not occur by modeling species’ distributions using MAXENT v.3.3.3 k [83]. MAXENT uses environmental and presence-only data to estimate the species’ current realized niche space, which is used to predict the suitability of surrounding habitat. The modeled species distributions were then converted into binary maps where genetic variation was only projected onto areas where the species were predicted to be present. The second step then maps environmentally-associated intraspecific variation across these inferred species distributions using the GDM framework [82].

The environmental variables included in both MAXENT and GDM modeling procedures were similar to those used previously [44]. These were comprised of a set of climate variables from the WorldClim database [84] at 30 arcsec resolution (~ 1 km), representing annual means, ranges, and seasonality in temperature and precipitation averaged over a 50-year period from 1950 to 2000 and a set of satellite remotely sensed variables comprising: a measure of vegetation biomass or surface moisture from the QuickScat archive for the year 2001 [85]; elevation from the Shuttle Radar Topography Mission (SRTM); percent tree cover, computed from the Vegetation Continuous Field (VCF) for the year 2001. To reduce cross-correlations between predictor variables, one of a pair of highly correlated variables (Pearson cross-correlations >0.9) was removed, keeping variables that were either more frequently used in previous studies, or that were easier to interpret. The resulting reduced set of environmental variables consisted of the following: BIO1 (annual mean temperature), BIO2 (mean diurnal range), BIO4 (temperature seasonality), BIO5 (maximum temperature of warmest month), BIO12 (annual precipitation), BIO15 (precipitation seasonality), BIO16 (precipitation of the wettest quarter), BIO17 (precipitation of the driest quarter), QSCATmean (mean annual QuickScat), QSCATStd (seasonality of QuickScat measurements), SRTM (elevation), Treecover (percent tree cover), NDVI Mean (mean annual Normalized Difference Vegetation Index - NDVI), NDVIStd (seasonality of NDVI), NDVIgr (NDVI in the greenest season), NDVIbr (NDVI in the brownest season), and NDVIgrbr (the difference in NDVI between greenest and brownest seasons). These environmental variables and their database sources are listed in Additional file 1: Table S2.

In addition to these environmental variables a set of predictor variables were generated to model the effect of Pleistocene refugia and rivers. A GIS layer was first generated for two refugia; one north of the Sanaga River in the Gulf of Guinea and another south of the Sanaga River encompassing southern Cameroon, Equatorial Guinea, and northern Gabon [12]. To create the GIS layer, the approximate center points of the refugia were coded as 0 and 1 respectively, and then an inverse distance weighted spatial interpolation in ArcGIS 9.3 was to compute a raster layer in which the influence of a refugium is highest closest to its centroid, but diminishes further away from it. This layer thus represented divergence between populations in the two refugia, with subsequent dispersal away from each refugium in all directions and at the same speed, allowing for admixture where dispersing individuals would have met. To model the effect of rivers, isolation-by-resistance (IBR) distances [86] were computed based on hypothesized riverine barriers. Resistance distances account for the differences in habitat permeability to compute a more realistic approximation of the distance an individual needs to travel from one population to the other relative to the straight-line geographic distance. Its advantage over least-cost path analysis is that it takes into account the fact that there often are multiple paths of low cost [87]. Resistance distances were generated for each species using CIRCUITSCAPE v.3.5.8 [88] and cost surfaces where perennial river systems with a Strahler number of 4, reflecting a high branching complexity, were defined as being impossible to cross. Grid cells in a GIS layer overlapping such river systems were allocated a high resistance of 1000, and all other cells were given a value of one.

The MAXENT model was fitted on all occurrence records (locations were at least 50 km apart and based on GPS data), within a study area bound by 16° W, 31° E, 10° N, and 10° S, thus incorporating the West and Central African rainforests and adjacent savanna regions. We used a convergence threshold of 10^−4^, a maximum of 500 iterations, 100 cross-validation replicates, maximum 10,000 background points, and default prevalence of 0.5. The default regularization multiplier of 1.0 resulted in species distributions that largely matched those based on expert opinion. A jackknifing procedure was performed to evaluate the importance of each environmental variable. The logistic output format was selected, which returns continuous values between 0 and 1 to indicate the environmental suitability of each grid square on the map. The predicted species distributions were mapped in ArcMap v.10.1 (ESRI, Redlands, CA), and cells with a minimum suitability threshold were deemed ‘unsuitable’. Minimum suitability was defined by the minimum training presence threshold, which was equal to 0.45, 0.47 and 0.01 for *C. dorsalis*, *C. callipygus*, and *P. monticola*, respectively. A presence-absence map was thus generated for use in subsequent GDM analyses, where models of genetic differentiation were only projected into areas of species’ presence. The maps were trimmed to include the region where the majority of sampling for this study was carried out, namely: Cameroon, Gabon, Equatorial Guinea and the Republic of Congo.

GDM modeling of each species was implemented in ArcView 3.2 and S-plus 4.0, and results were visualized in ArcGIS 9.3 (ESRI, CA). The importance of each predictor variable was tested by iteratively adding and removing each variable to the model and comparing the variation explained to other such models. Only predictors that made a significant contribution to explaining variation in the response variable were retained. Overall model performance was evaluated relative to a null model based on random environmental variables. Competing models were thus constructed with the following sets of predictor variables: 1) environment + refugia + geographic distance; 2) environment + resistance distance + geographic distance; 3) environment + geographic distance; 4) environment; 5) refugia; 6) resistance distance; 7) geographic distance; 8) random environmental variables (i.e. null model).

The resulting function from the best-fit model describing the relationship between genetic differentiation and the significant predictor variables was used in a subsequent step to interpolate patterns of genetic differentiation across the entire study area. For visualization purposes in the resulting maps, areas were color-coded, where the magnitude of the color difference between two locations represents the magnitude of genetic differentiation.

## Results

### Species identification

Of the 2040 collected fecal samples, a total of 1146 were identified to species level, leaving 894 that failed to amplify. The identity of all museum and reference tissue samples was also confirmed by our control region diagnostic. As expected, three species were well represented in our fecal collections and were subsequent targets for the present study: *C. dorsalis*, *C. callipygus* and *P. monticola*. Five other species were also identified: the yellow-backed duiker *C. silvicultor,* the black-fronted duiker *C. nigrifrons,* the white-bellied duiker *C. leucogaster*, the water chevrotain *Hyemoschus aquaticus* and the sitatunga *Tragelaphus spekii*. However, the low sample sizes in these species precluded them from any further analyses (Table 1)*.* STRUCTURE was able to unambiguously identify *P. monticola* samples from NGO but could not distinguish between other sympatric duiker species (Additional file 2: Figure S1). As a result, only *P. monticola* samples from the NGO site were incorporated into further analyses.

**Table 1:** Sampling locations, sites and number of samples identified to species level at each site

Country	Number of sites	*C. dorsalis*	*C. callipygus*	*P. monticola*	*C. silvicultor*	*C. nigrifrons*	*C. leucogaster*	*H. aquaticus*	*T.* *spekii*	Total
Cameroon	14	39	37	81	2	0	0	2	2	177
Central African Republic	1	4	0	15	0	0	0	0	0	20
Democratic Republic of Congo	2	1	5	2	3	0	0	0	0	13
Equatorial Guinea	2	6	6	37	0	1	0	0	0	52
Gabon	14	72	360	165	31	1	14	67	37	761
Nigeria	1	0	6	35	0	0	0	0	0	42
Republic of Congo	5	6	61	33	3	3	2	2	0	115
Republic of Guinea	1	2	0	0	0	0	0	0	0	3
South Africa	1	0	0	1	0	0	0	0	0	2
Unknown	1	1	0	1	0	0	0	0	0	3
Total	42	131	475	370	39	5	16	71	39	1146

### Mitochondrial DNA sequencing and microsatellite genotyping

Owing to the high amount of variation in the control region dataset, sequences were collapsed into composite haplotypes prior to construction of haplotype networks. This collapse step left 16 composite haplotypes for *C. dorsalis* (sequences differing at ≤17 nucleotide positions), 27 for *C. callipygus* (sequences differing at ≤27 nucleotide positions), and 25 for *P. monticola* (sequences differing ≤31 nucleotide positions). Within *C. dorsalis*, most haplotypes were widespread across the study area except for haplotypes K-P which were restricted to single sites (Additional file 2: Figure S2a and Additional file 1: Table S3). Similarly, *C. callipygus* did not reveal any obvious pattern of genetic structure, although haplotypes P, Q, S, T, V, X, Y, Z and Φ were also unique to specific sites (Additional file 2: Figure S2b and Additional file 1: Table S4). Lastly, within *P. monticola*, most of the NIG samples formed a cluster (haplotypes J, N, and R) that was highly differentiated from other sequences in the dataset (Additional file 2: Figure S2c and Additional file 1: Table S5). Phylogenetic analysis of mitochondrial COI sequences showed that these samples are from *P. walteri*, the new duiker species described by [71] (Additional file 2: Figure S3). The average pairwise Tamura-Nei distances (Additional file 3) were lowest between candidate *P. walteri* samples from NIG and diagnosed *P. walteri* samples (average 0.012 base substitutions/site) and highest when compared to *P. monticola* (average 0.111 base substitutions/site). These sequences were removed from any further mitochondrial DNA analyses. Other haplotypes within this species were also widespread with the exception of P, Q, S, U, W, X, and Y which were restricted to individual sites. Two haplotypes (T, V) restricted to south-eastern Gabon and Equatorial Guinea respectively both possessed a 77 bp indel. The highest mitochondrial haplotype diversity in *C. dorsalis* was found on the east bank of the Ivindo River in central Gabon (IV_E_), southeastern (LBK), central (MBJ) and south-western Cameroon (TAK), and the Ngotto region of the Central African Republic (NGO). The highest average nucleotide diversity (π) and highest polymorphism per site (θ) was found in LBK (Additional file 1: Table S6). For *C. callipygus*, the highest haplotype diversity was found in Bioko Island (BKO) and central D.R.C. (SA). In contrast, π and θ was greatest on BKO (Additional file 1: Table S6). For *P. monticola*, the highest haplotype diversity was found on the east and west banks of the Ivindo River in central Gabon (IV_E_, IVw) and south-eastern Cameroon (LBK), whereas π and θ was greatest in south-western Cameroon (EBO) (Additional file 1: Table S6).

With respect to the microsatellite data, we detected very few instances of deviations from HWE (Additional file 1: Tables S7–9) or LE (Additional file 1: Table S10) after correcting for table-wise error. GIMLET analyses revealed that the minimum number of typed microsatellite loci needed to identify first-order relatives was five for *C. dorsalis*, six for *C. callipygus,* and four for *P. monticola* (Additional file 1: Table S11). KINGROUP analyses found 11 out of 65 (*C. dorsalis*), 77 out of 310 (*C. callipygus*), and 82 out 332 (*P. monticola*) samples were first-order relatives of other individuals in the dataset. These individuals were subsequently removed from all further analyses.

### Pleistocene refugia hypothesis

There was no evidence for any obvious mitochondrial genetic structure within *C. dorsalis* (Table 2, Additional file 1: Table S12), although SAMOVA uncovered a significant among group variance component (F_CT_ = 16.31%; *P* = 0.014) for three clusters comprising a riverine site in Cameroon (NKO), Gabon (OLO_S_) and the rest of the dataset. SAMOVA analysis of *C. callipygus* identified a significant among group variance component (F_CT_ = 18.25%; *P* = 0.007) comprising samples from the Gulf of Guinea refugium (NIG, TAK), central D.R.C. (SA) and all other sites. Partitioning the dataset into two groups comprising only the Gulf of Guinea refugium and all remaining sites also revealed a marginally significant among group variance component (Table 2, Additional file 1: Table S13). Similarly, SAMOVA analysis of *P. monticola* identified a high among group variance component (F_CT_ = 36.831%; *P* = 0.010) for three clusters comprising Gulf of Guinea refugium (NIG, TAK, BAM), Sanaga River mouth (EDEA) and elsewhere across the study region. Once more, partitioning the dataset into the Gulf of Guinea refugium versus all other sites revealed an among group component that was only marginally significant (Table 2, Additional file 1: Table S14). There was no evidence of significant genetic structure when samples were clustered by additional candidate refugia except in the case of *P. monticola* when a significant among group variance component was detected (F_CT_ = 6.41%, *P* = 0.019) when sites were grouped according to the Sanaga River basin (Table 2, Additional file 1: Table S14). Lastly, a weak riverine barrier effect (F_CT_ = 10.02%, *P* = 0.024) was observed in *P. monticola*, but only when sites were grouped according to both the Sanaga and the Ogooué rivers.

**Table 2:** The among group component of the total variance (F_CT_) for hypothesized *C. dorsalis, C. callipygus, and P. monticola* groupings

Models	*C. dorsalis*	*C. callipygus*	*P. monticola*
Gulf of Guinea refugium versus all other sites	−0.026 (2)	0.195* (2)	0.283‡ (2)
Central African refugia versus all other sites	0.027 (4)	0.055 (4)	−0.008 (5)
Sanaga fluvial refugium versus all other sites	0.000 (2)	−0.012 (2)	0.064* (2)
Ogooué River barrier: Ogooué north versus Ogooué south	0.145 (2)	−0.012 (2)	−0.034 (2)
Sanaga River barrier: Sanaga north versus Sanaga south	−0.026 (2)	NT	−0.103 (2)
Sanaga and Ogooué River barriers: Sanaga north versus Sanaga south and Ogooué North versus Ogooué south	0.008 (3)	−0.003 (3)	0.100* (3)

Numbers in parenthesis indicate the number of groups for each tested hypothesis

Asterisk indicates significance levels: ‡ marginally significant (*P* < 0.10); *significant (*P* < 0.05)

NT: not tested

In *P. monticola*, both π (U-value = 11, *P* = 0.011) and θ (U-value = 9.5, *P* = 0.006) was significantly higher in refugial versus non-refugial sites. A significant negative Fu’s Fs statistic was also observed in the largest SAMOVA grouping of sites for *C. dorsalis* (Fs = −24.04, *P* = 0.011), *C. callipygus* (F_S_ = −23.44, *P* = 0.006) and *P. monticola* (F_S_ = −23.59, *P* = 0.009) and is consistent with a history of demographic expansion across the entire study area. A significant signature of expansion was also observed in the Sanaga River basin grouping for *C. dorsalis* (Fu’s F_S_ = −5.04, *P* = 0.02), *C. callipygus* (Fu’s F_S_ = −3.86, *P* = 0.036) and *P. monticola* (Fu’s Fs = −24.41, *P* < 0.001).

STRUCTURE analyses of *C. dorsalis* indicated that the best estimate of K = 2 although there was no obvious geographic pattern in the dataset (Additional file 2: Fig. S4a-b). For *C. callipygus*, there was no obvious geographic pattern and the best K could not be reliably determined (Additional file 2: Fig. S5a-b). However, the raw mean estimated likelihood probability of data at K = 1 was not the highest, indicating that a model of population subdivision was more plausible than one single panmictic population*.* For *P. monticola*, the best K = 2 with NGO constituting one population and all other sites falling into the other (Additional file 2: Fig. S6a-b). Lastly, GDM analyses revealed no effect of a Pleistocene refugium in any of the three species datasets (Table 3).

**Table 3:** Percentage of genetic differentiation explained in the GDM models

Model	Percentage of genetic differentiation explained		
	*C. dorsalis*	*C. callipygus*	*P. monticola*
Random variables (null model)	83.06	8.35	20.82
Geographic distances only	54.00	11.96	17.33
IBR distances only	63.22	6.80	13.92
Hypothesized refugia only	63.62	0.01	0.09
Significant environmental variables only^a^	81.25 (QSCATStd, NDVIbr, NDVIme)	60.13 (QSCATStd, QSCATmax, NDVImax, Bio 04, Bio 02, NDVIgr, Bio 01, Bio 12)	72.85 (QSCATstd, QSCATmax, Bio 17, Bio 05, Bio 16, Tree Cover),
Significant environmental variables^a^ and geographic distances	81.25	60.87	72.68
Significant environmental variables, geographic distance and IBR distances	83.18	60.87	72.68
Hypothesized refugia with significant environmental variables and geographic distance	81.25	60.87	73.88

^a^See Additional file 1: Table S2 for explanation of environmental variables

### Riverine barriers

Overall both AMOVA and STRUCTURE analyses did not provide evidence for an effect of a riverine barrier. However, there was weak support for a riverine effect in *P. monticola*, but only when sites were partitioned according to both the Sanaga and the Ogooué rivers. GDM analyses using a resistance distance-based approach also revealed no effect of a riverine barrier in any of the three species (Table 3).

### Landscape genetic models of genetic variation

The AUC values of the MAXENT species distribution models suggested high model performance for each of the three species (AUC > 0.930 for all models). Environmental variables from either WorldClim (Additional file 2: Figure S8) or satellite QSCAT and MODIS sources demonstrate considerable heterogeneity at the landscape scale (Additional file 2: Fig. S7a and b). The variables that were identified as having the strongest influence on species’ distributions were surface moisture/canopy roughness (QSCATmean and QSCATStd) for *C. dorsalis* and *C. callipygus*, and mean diurnal temperature range (Bio 02) for *P. monticola* (Additional file 2: Fig. S8–10). Both mean diurnal temperature range (Bio 02) and temperature seasonality (Bio 04) were also important predictors for *C. dorsalis* and *C. callipygus*, as was QSCATmean and QSCAStd for *P. monticola*. Precipitation in the driest quarter (Bio 17) and tree cover provided unique information for *C. dorsalis* and *C. callipygus* respectively, whereas annual precipitation (Bio 12) and precipitation of the wettest quarter (Bio 16) provided unique information for *P. monticola.* Although all species were predicted to occur across much of the study area, the distribution of *C. callipygus* and *C. dorsalis* was patchy across Cameroon and Equatorial Guinea whereas for *P. monticola*, it was relatively uniform (Additional file 2: Fig. S11).

For *C. dorsalis*, a model with random environmental variables explained as much of the observed genetic differentiation as the best performing model based on environmental variables, geographic distance and resistance distances (Table 3). We therefore do not report further results for this species. In contrast, environmental variables explained the highest proportion of genetic differentiation in both *C. callipygus* and *P. monticola* models, suggesting that environmental heterogeneity is a main driver of population divergence in both species (Table 3). Unlike *C. dorsalis*, random models for *C. callipygus* and *P. monticola* explained relatively little of the variation in comparison to models that included significant environmental predictors. Geographic distance had little to no effect on model performance, performing only slightly better than the random model for *C. callipygus* and no better than random for *P. monticola*. The addition of resistance distances as a proxy for a riverine barrier effect did not improve model fit either and models based on resistance distances alone explained even less variation than the model based on only straight-line geographic distance. Including refugia did not improve the explanatory power of the model for *C. callipygus* and improved it only incrementally for *P. monticola.* Models with refugia as the only predictor variable explained little if any variation.

**Fig. 2:**
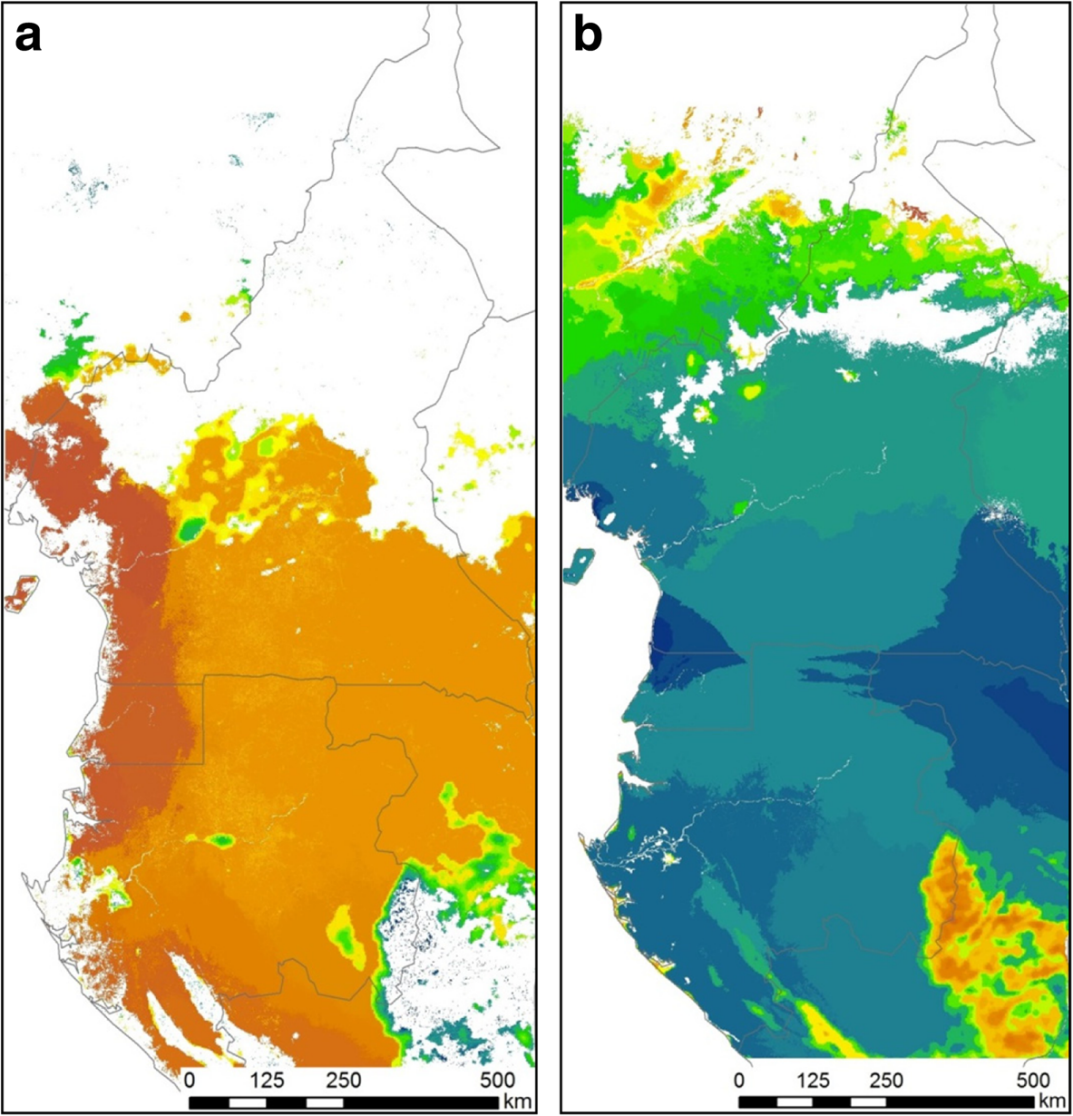
Maps of the generalized dissimilarity models for a) *C. callipygus* and b) *P. monticola*. Pairwise comparison of colors between any two points in the landscape indicates the genetic differentiation between those points: larger color differences correspond to larger genetic differences. Areas in white indicate where the species is not predicted to occur

As was the case in MAXENT, one of the most important environmental variables for predicting patterns of genetic differentiation in *C. callipygus* and *P. monticola* was QSCAT_mean_ and QSCAT_std_, which in this area is an indication of vegetation biomass. Green leaf vegetation (NDVImax and NDVIgr) and temperature variables (Bio 01, Bio 02, Bio 04 and Bio 12) were all significant in explaining observed genetic differentiation in *C. callipygus* (Additional file 2: Fig. S12) whereas precipitation variables (Bio 16 and Bio 17), percent tree cover and the maximum temperature of the warmest month (Bio 05) were significant predictors for *P. monticola* (Additional file 2: Fig. S13). For both *C. callipygus* and *P. monticola,* areas of pronounced turnover in environmentally-associated genetic differentiation were found in the montane areas of the Gulf of Guinea and across the Batéké Plateaux region between Gabon and the Congo (Fig. 2). Genetic turnover was also more pronounced between the coast and interior portion of the study area for *C. callipygus* whereas a more subtle shift towards the interior of the Congo Basin was evident in *P. monticola*.

## Discussion

The present study is one of the first to simultaneously assess the roles of Pleistocene refugia, riverine barriers, isolation by distance and environmental heterogeneity in shaping the diversification of central African forest duiker species. While analyses of mitochondrial structure suggest that the Gulf of Guinea may have constituted a historical refugium for at least two of three of the species studied here, subsequent population expansion during the warmer inter-glacial periods has erased any signature of population structure in nuclear microsatellite markers. Unlike several vertebrate species that have been studied to date [17, 19, 45], the effect of riverine barriers appears negligible in rainforest duikers. GDM analyses of nuclear microsatellite data confirm that neither historical forest refugia nor rivers play an important role and that population structure is instead driven by environmental heterogeneity.

These findings have important implications for the study of central African phylogeography since the impact of environmental variation on current population structure is rarely considered [30, 44]. Few studies have attempted to evaluate the relative importance of competing hypotheses of diversification within a comparative framework, and of those that have, most have focused on purely vicariant models of diversification [19, 89–92]. Here we summarize our main findings with respect to each hypothesized mode of diversification and conclude with a perspective on their implications for the conservation of central African biodiversity.

### Pleistocene refugia

SAMOVA analyses indicate samples of *C. callipygus* and *P. monticola* from the Gulf of Guinea sites are modestly differentiated from most other sites sampled across their range, providing some support for this region as a historical refugium. Phylogenetic studies suggest that Pleistocene refugial dynamics have played an important historical role in shaping the speciation of this group [93] although subsequent population expansion has likely dampened historical genetic structure. The Gulf of Guinea is known to be an important center of species diversity and endemicity [94–97] and harbors distinct lineages of several rainforest taxa [19, 32, 44, 91, 98, 99] including that of *P. walteri*. Such lineages are likely to have arisen as a result of Pleistocene forest fragmentation, providing corroborating support for this region as a driver of diversification.

However, unlike studies on the western *Gorilla gorilla gorilla* [17], rainforest tree *Aucoumea klaineana* [100, 101] and marsh rat *Malacomys longipes* [32], hypothesized refugia in southwestern Cameroon, Equatorial Guinea and Gabon do not appear to have played a significant role in shaping duiker regional genetic structure. Instead, all three duiker species exhibit signatures consistent with a history of a post-glacial expansion across much of the study area. This signature of historical expansion has also been observed in several other co-distributed taxa that are relatively flexible in their use of habitat [99] or have relatively high dispersal capabilities [90, 102]. Our findings also suggest that duikers may have been able to cross savanna-forest mosaic environments during periods when forests were either fragmented or undergoing postglacial expansion and that this expansion might have been so extensive as to erase any signatures of historical population structure. Field observations in a naturally fragmented landscape in central Gabon provide some support to this hypothesis [103] and suggest that although duikers are generally tied to forest habitats, they are able to cross intervening savanna areas to gain access to forest fragments or even inhabit thicket habitats [56].

Several previous studies have also suggested that river basins [26] or gallery forests flanking smaller rivers and streams [27, 104] may have constituted fluvial refugia during the dry phases of the Pleistocene. AMOVA analysis of mitochondrial datasets does provide limited support for this hypothesis in showing that *P. monticola* populations within the Sanaga River basin are genetically differentiated from the surrounding area and that all three taxa exhibit a signature of population expansion. STRUCTURE analyses also indicate that *P. monticola* populations in the NGO area of southern Central African Republic are differentiated from the rest of the study region, possibly due to a riverine refugium within the Sangha River basin [17]. However, more detailed sampling is needed to evaluate this hypothesis more fully.

It is also important to note that the mitochondrial break between the Gulf of Guinea refugium and the rest of the study area was not observed in the nuclear microsatellite dataset and that refugia added little or nothing to the microsatellite-based GDM model. This discrepancy is probably due to the fact that mitochondrial data is more likely to reflect historical genetic structure than microsatellites, which in turn are better able to capture contemporary drivers of genetic structure. These differences could also be due to the lower genetic effective population size of mitochondrial DNA and/or greater female philopatry of duikers [57].

### Riverine barriers

Findings from this study do not support a role for the Sanaga and Ogooué rivers as barriers to dispersal in duikers. The only possible exception is *P. monticola* where AMOVA analyses suggest a weak pattern of differentiation between rivers. However, this result might also be an artifact created by structuring due to the Sanaga River basin. The lack of a strong riverine barrier effect could be surprising as both the Sanaga and Ogooué rivers are known to be important biogeographical barriers in other species [17, 38, 105]. Although the length and water flow rate of the Ogooué (920 km and 4645 m^3^/s) and Sanaga (890 km, 3100 m^3^/s) rivers are considerable [106], other factors such as the history, seasonality, and channel dynamics during the dry phases of the Pleistocene [107] may have allowed duikers to cross them more readily than has previously been supposed [57].

### Environmental heterogeneity

GDM analyses reveal that environmental variation explains a large proportion of genetic differentiation in both *C. callipygus* and *P. monticola*. Remarkably, most of the genetic differentiation between sites can be explained by a handful of environmental variables associated with temperature, moisture and vegetation cover. Interestingly, previous GDM analyses of neutral variation in chimpanzee *Pan troglodytes* subspecies [45] and the rainforest skink *Trachylepus affinis* [44] have shown that population genetic structure is also shaped by a similar set of environmental variables, emphasizing their importance as predictors of suitable habitat and contemporary population structure across the region.

The present study also indicates that the greatest turnover in environmentally-associated variation is found in the Gulf of Guinea and in the Plateaux Batéké region of southeastern Gabon. Both regions encompass important environmental and/or elevational gradients that would be expected to play a major role in diversification. Previous GDM studies on chimpanzees and skinks mirror elevated genetic turnover in the Gulf of Guinea region and also suggest a strong east-west pattern of genetic differentiation from the coast to the interior [44, 45]. However, as sampling in both studies was limited to Cameroon, it is impossible to compare patterns of differentiation further south and along the Equator.

The narrow coastal to interior pattern of genetic differentiation observed in *C. callipygus* is likely to reflect the east-west gradient in annual precipitation (Bio 12) commonly experienced across this region [108]. Although previous comparative phylogeographic studies in rainforest trees have so far failed to find evidence for this gradient [30], findings from *C. callipygus* would appear to support this hypothesis. In *P. monticola*, we also observed a modest shift in genetic structure between the western portion of the study area and the interior of the Congo basin that mirrors shifts in the distribution of precipitation in the driest quarter (Bio 17). Taken together, these findings clearly demonstrate that a GDM approach can uncover cryptic genetic structure that would have otherwise been missed using more conventional landscape genetic approaches and identify which environmental variables might be the most important drivers of genetic differentiation.

### Ecological differences between species

Although mitochondrial data indicate that the Gulf of Guinea refugium has played a role in structuring *C. callipygus* and *P. monticola* populations, this pattern was not evident in *C. dorsalis*. This effect could be simply due to limited number of samples obtained for *C. dorsalis* within the Gulf of Guinea refugium or due its wider geographic distribution [56], larger home range [58], and broad dietary flexibility [109] that may have made it less sensitive to past forest refugial dynamics. Nevertheless, all three species exhibit signatures of past population expansion, indicating a common demographic history. Future work should include sampling from candidate refugia in West Africa [12] in order to better assess the impact of Pleistocene history on *C. dorsalis*.

Of the three focal taxa in this study, *P. monticola* appears to be the species whose genetic structure is most likely to have been affected by fluvial refugia. *P. monticola* has a much smaller home range than either of the other two other species in this study [57] and consequently may have been able to better persist in more restricted habitats such as gallery forest than other duikers. The subtle shift in genetic differentiation towards the Congo basin for this species may also reflect an important transition from lowland to swamp forest [110]. *C. callipygus* is the only species to show a pronounced pattern of genetic differentiation from the coast to the interior that could either reflect its greater sensitivity to rainfall gradients and/or be due to the fact that the distribution of its sister taxon *C. ogilbyi* is along the coast. Interpretation of these findings is complicated even more by the fact that genetic markers cannot distinguish *C. ogilbyi* from *C. callipygus* [62, 93]. Future work should aim to increase sampling of known individuals of both species in order to better assess their genetic structure and taxonomic status.

## Conclusions

The rainforests of central Africa have attracted considerable attention because of their remarkable species richness and endemism [23, 111, 112], high economic value [113], and global importance as a carbon sink [114]. However, our understanding of the causes of biological diversification and the landscape elements that promote these processes remains poor. This has been compounded by the fact that evolutionary research on cryptic rainforest vertebrates such as forest duikers has traditionally been very challenging, requiring geo-referenced non-invasive sampling and intensive genotyping technologies such as those employed here [115].

Building on our previous phylogenetic analysis of the Cephalophinae [93], this study points to a modest role for the Gulf of Guinea refugium in shaping mitochondrial genetic structure. As predicted under the Pleistocene refugia hypothesis, signatures of population expansion are also evident across all three taxa and are consistent with a history of post-refugial expansion. GDM of the microsatellite data also points to the importance of environmental heterogeneity in shaping current patterns of genetic structure. Regions of strong environmental transition may constitute important drivers of evolutionary change and as such merit further conservation attention. Future climate change may also lead to shifts in the location and persistence of such environmental gradients, underlining the need to incorporate forward projections into both species distribution modeling and the GDM frameworks presented here. This may also reveal differences in the sensitivities of different taxa to shifts in the distribution of suitable habitat which will be important for conservation planning purposes [116].

Although the taxonomic scope of the present study is still very limited, it is clear that as a forest interior specialist, *C. callipygus* is likely to be much more sensitive to habitat changes than *P. monticola*. It therefore appears urgent that more studies are carried out in this region to better understand not only how historical climate change and environmental heterogeneity have shaped past and present population structure but also how future landscape modification and climate change will ultimately affect species’ capacity to adapt or move under different development scenarios [117].

## Additional files

Additional file 1: Supplementary tables. **Table S1**. Sampling locations by country, site name, letter code, and corresponding sample sizes for identified species in our study area. **Table S2.** Summary of environmental variables used in MAXENT and GDM models. **Table S3.** Distribution of composite *C. dorsalis* haplotypes by site. **Table S4.** Distribution of composite *C. callipygus* haplotypes by site. **Table S5.** Distribution of composite *P. monticola* haplotypes by site. **Table S6.** Mitochondrial diversity indices for each species by site. **Table S7.** *C. dorsalis* expected heterozygosity and tests of deviation from Hardy-Weinberg Equilibrium. **Table S8.** *C. callipygus* expected heterozygosity and tests of deviation from Hardy-Weinberg Equilibrium. **Table S9.** *P. monticola* expected heterozygosity and tests of deviation from Hardy-Weinberg Equilibrium. **Table S10.** Significant associations between pairs of loci for *C. callipygus* and *P. monticola*. **Table S11.** Probability of identity (PI_sibs_) for each locus (within species) and cumulative probabilities of identity across multiple loci for each species. **Table S12.** The among group component of the total variance (F_CT_) for hypothesized *C. dorsalis* groupings. **Table S13.** The among group component of the total variance (F_CT_) for hypothesized *C. callipygus* groupings. **Table S14.** Among group component of the total variance (F_CT_) for hypothesized *P. monticola* groupings (DOCX 113 kb)

Additional file 2: Supplementary figures. **Figure S1**. Graphical representation of the STRUCTURE output for the identification of unknown NGO samples for K = 2. **Figure S2a.** minimum Spanning Network (MSN) of *C. dorsalis* based on 16 composite haplotypes (collapsed haplotypes). Red circles indicate the median vector or hypothesized (ancestral) haplotypes and the length of each branch connecting sampled haplotypes indicates the genetic distance between haplotypes. b. Minimum Spanning Network (MSN) of *C. callipygus* based on 27 composite haplotypes (collapsed haplotypes). *C. minimum* Spanning Network (MSN) of *P. monticola* based on 25 composite haplotypes (collapsed haplotypes). **Figure S3.** Cytochrome c oxidase subunit I neighbour-joining bootstrap consensus phylogeny based on Tamura-Nei distances. Bootstrap values of 75% or greater are indicated at the relevant node. **Figure S4a-b.** Graphical representation of the STRUCTURE output for *C. dorsalis* for K = 2. **Figure S5a-b.** Graphical representation of the STRUCTURE output for *C. callipygus* for K = 6. **Figure S6a-b.** Graphical representation of the STRUCTURE output for *P. monticola* for K = 2. **Figure S7.** Environmental variables used as predictors in MAXENT and GDM analyses. **Figure S8.** Jackknife of regularized training gain for *C. dorsalis*. **Fig. S9**. Jackknife of regularized training gain for *C. callipygus*. **Figure S10.** Jackknife of regularized training gain for *P. monticola*. **Figure S11.** MAXENT predicted species distributions for: a) *C. dorsalis* b) *C.callipygus* and c) *P. monticola*. **Figure S12.** GDM functional response curves for *C. callipygus*. **Figure S13.** GDM functional response curves for *P. monticola (PPTX 2695 kb)*

Additional file 3: Matrix of DNA distances. (XLS 48 kb)
