# Supplementary material for: Evaluating the role of Pleistocene refugia, rivers and environmental variation in the diversification of central African duikers (genera Cephalophus and Philantomba)
Source: BMC Evol Biol. 2017 Sep 6;17:212. doi: 10.1186/s12862-017-1054-4 (PMC5585889; doi:10.1186/s12862-017-1054-4)
Supplement: Supplementary file 1 — Supplementary tables. Table S1. Sampling locations by country, site name, letter code, and corresponding sample sizes for identified species in our study area. Table S2. Summary of environmental variables used in MAXENT and GDM models. Table S3. Distribution of composite C. dorsalis haplotypes by site. Table S4. Distribution of composite C. callipygus haplotypes by site. Table S5. Distribution of composite P. monticola haplotypes by site. Table S6. Mitochondrial diversity indices for each species by site. Table S7. C. dorsalis expected heterozygosity and tests of deviation from Hardy-Weinberg Equilibrium. Table S8. C. callipygus expected heterozygosity and tests of deviation from Hardy-Weinberg Equilibrium. Table S9. P. monticola expected heterozygosity and tests of deviation from Hardy-Weinberg Equilibrium. Table S10. Significant associations between pairs of loci for C. callipygus and P. monticola. Table S11. Probability of identity (PIsibs) for each locus (within species) and cumulative probabilities of identity across multiple loci for each species. Table S12. The among group component of the total variance (FCT) for hypothesized C. dorsalis groupings. Table S13. The among group component of the total variance (FCT) for hypothesized C. callipygus groupings. Table S14. Among group component of the total variance (FCT) for hypothesized P. monticola groupings (DOCX 113 kb) [file 12862_2017_1054_MOESM1_ESM.docx]

Table S1. Sampling locations by country, site name, letter code, and corresponding samples size for identified species in our study area (see Figure 1).

| Country | Site names | Site code | Sample size | | | | | | | | | |  |
| --- | --- | --- | --- | --- | --- | --- | --- | --- | --- | --- | --- | --- | --- |
|  |  |  | *Ceph.*  *dorsalis* | *Ceph. callipygus* | *Phil.*  *monticola* | *Ceph.*  *silvicultor* | *Ceph nigrifrons* | *Ceph leucogaster* | *Hyem.*  *aquaticus* | *T.rag.*  *spekei* | *Phil.*  *maxwelli* | *Phil.*  *walteri* | |
| Cameroon | Campo Reserve | CPO | 2 | 12* | 21* | - | - | - | - | - | - | - | |
| Cameroon | Deng Deng | DENG | - | - | 3* | - | - | - | - | - | - | - | |
| Cameroon | Bamenda | DIV | - | - | 1 | - | - | - | - | - | - | - | |
| Cameroon | Dja Reserve | DJA | 3 | - | - | - | - | - | - | - | - | - | |
| Cameroon | Ebo Forest | EBO | 9* | 1 | 19* | - | - | - | 1 | - | - | - | |
| Cameroon | Douala-Edea Forest Reserve/Ekoth | EDEA | - | - | 8* | - | - | - | - | 2 | - | - | |
| Cameroon | Ekanga/Masseng/  Biwali/Ngamba Enduum | EKA | - | - | 3* | - | - | - | - | - | - | - | |
| Cameroon | Kombe | KOM | 2* | 1* | 5* | - | - | - | - | - | - | - | |
| Cameroon | Parc National de Lobéké | LBK | 5* | 16* | 4 | 1 | - | - | - | - | - | - | |
| Cameroon | Linté/Ngambe Tikar | LIN | 1* | - | - | 1 | - | - | - | - | - | - | |
| Cameroon | Parc National de Mbam et Djerem/Wouchaba | MBJ | 4* | 5* | - | - | - | - | 1 | - | - | - | |
| Cameroon | Nkolomaken | NKO | 5* | - | 6* | - | - | - | - | - | - | - | |
| Cameroon | Lom/Pangar | PAN | 2* | - | 1* | - | - | - | - | - | - | - | |
| Cameroon | Takamanda Forest Reserve | TAK | 6* | 2 | 10* | - | - | - | - | - | - | - | |
| Central African Republic | Ngotto zone Mbaéré-Bodingué National Park | NGO | 4 | - | 15* | - | - | - | - | - | - | - | |
| Democratic Republic of Congo | Kisangani | KIS | - | - | 1 | - | - | - | - | - | - | - | |
| Democratic Republic of Congo | Parc National de Salonga | SA | 1 | 5* | 1 | 3 | - | - | - | - | - | - | |
| Equatorial Guinea | Reserva Científica de la Caldera de Luba, Bioko | BKO | - | 5* | 2 | - | 1 | - | - | - | - | - | |
| Equatorial Guinea | Monte Mitra/Monte Alen | MM/  MTA | 6* | 1 | 35* | - | - | - | - | - | - | - | |
| Gabon | Gamba, Iguela, Malounga | GA | - | 2 | 33* | 3 | - | - | 15 | - | - | - | |
| Gabon | Boumango, Parc National des Plateaux Batéké, Leconi, Bakoumba, Ossélé/Kessala | HAO | 3 | 15* | 7* | 2 | - | 4 | 9 | 1 | - | - | |
| Gabon | Ivindo/Ipassa/  Dji dji (West and East) | IV_W_, IV_E_ | 8* | 29* | 17* | 3 | - | 1 | 4 | - | - | - | |
| Gabon | Langoué | LA | 9* | 21* | 6* | 1 | 1 | 4 |  | - | - | - | |
| Gabon | Parc National de la Lopé (north and south) | LO_N_, LO_S_ | 1 | 143* | 40* | 10 | - | 1 | 18 | 1 | - | - | |
| Gabon | Parc National des Monts de Cristal | MCR | 12* | 1 | 16* | - | - | - | 1 | - | - | - | |

| Gabon | Parc National de Birougou | MFCH | 5* | 8* | 9* | - | - | 1 | - | - | - | - |
| --- | --- | --- | --- | --- | --- | --- | --- | --- | --- | --- | --- | --- |
| Gabon | Parc National Minkébé | MKB | 3 | 11* | 4 | 2 | - | 1 | - | - | - | - |
| Gabon | Moyen Ogooué (north and south) | MOO_N_, MOO_S_ | - | - | 6* | 6 | - | - | 12 | 23 | - | - |
| Gabon | Mitzic | MTZ | - | 1 | - | - | - | 1 | - | - | - | - |
| Gabon | Ogooué Ivindo (north and south) | OIV_N_, OIV_S_ | 9* | 54* | 7* | 1 | - | - | 5 | - | - | - |
| Gabon | Ogooué Lolo (north and south) | OLO_N_, OLO_S_ | 6* | 46* | 11* | 3 | - | 1 | 3 | - | - | - |
| Gabon | Ogooué Maritime | OMA | 1 | - | 1 | - | - | - | - | 12 | - | - |
| Gabon | Parc National de Waka | WA | - | 11* | 1 | - | - | - | - | - | - | - |
| Nigeria | Mbe, Oban, and Afi | NIG | - | 6* | 35* | - | - | - | - | - | - | - |
| Republic of Congo | Brazzaville area | BRA | - | 1 | - | - | - | - | - | - | - | - |
| Republic of Congo | Reserve de la Lefini | LEF | 2 | - | 1 | - | - | - | - | - | - | - |
| Republic of Congo | Parc National de Nouabelé Ndoki | ND/CO | 1 | 60* | 30* | 3 | 3 | 2 | 2 | - | - | - |
| Republic of Congo | Parc National d’Odzala | ODZ | 1 | - | - | - | - | - | - | - | - | - |
| Republic of Guinea | Diecke | DIE | 2 | - | - | - | - | - | - | - | - | - |
| South Africa | Cape province | CAP | - | - | 1 | - | - | - | - | - | - | - |
| Gabon | Unknown† | None | 15 | 18 | 8 | 1 | - | - | - | - | - | - |
| Republic of Congo | Unknown† | None | 6 | 7 | 8 | 3 | 6 | 5 | - | - | - | - |
| Cameroon | Unknown† | None | - | 2 | - | - | - | - | - | - | - | - |
| Benin | Unknown† | None | - | - | - | - | - | - | - | - | - | 3 |
| Unknown† | Unknown† | None | 1 | - | 30 | 1 | - | - | - | - | 3 | - |
| **Total** |  |  | 135 | 484 | 405 | 43 | 11 | 21 | 71 | 39 | 3 | 3 |

*Numbers with asterisk indicate samples that were also genotyped with 11 polymorphic microsatellites.

†Note that reference samples of unknown location but known species identity were used to identify NGO fecal samples.

Table S2. Summary of environmental variables used in MAXENT and GDM models.

| Type of variable | Database | Data source | Variables |
| --- | --- | --- | --- |
| Bioclimatic | Worldclim  [www.worldclim.org](http://www.worldclim.org/) | Ground-based weather stations | Bio 01 = Annual mean temperature  Bio 02 = Mean diurnal temperature range  Bio 04 = Temperature seasonality  Bio 05 = Max temperature of warmest month  Bio 12 = Annual precipitation  Bio 15 = Precipitation seasonality  Bio 16 = Precipitation of wettest quarter  Bio 17 = Precipitation of driest quarter |
| Vegetation biomass (inferred through surface moisture and canopy roughness) | QuickScat  <http://www.scp.byu.edu/data/Quickscat/SIRv2/qush/World_regions.htm> | Satellite-QSCAT | QSCATmean  QSCATStd |
| Topography, elevation | DEM  <http://srtm.csi.cgiar.org/> | Satellite-SRTM | SRTM |
| Forest cover | Percent tree cover  <http://landcover.org/data/vcf> | Satellite-MODIS | Tree cover |
| Green leaf vegetation | NDVI (Normalized Difference Vegetation Index)  <http://asterweb.jpl.nasa.gov/data.asp> | Satellite-MODIS | NDVIMean, NDVIStd, NDVIgr, NDVIbr, NDVIgrbr |

Table S3. Distribution of composite *C. dorsalis* haplotypes by site.

| **Site codes** | A | B | C | D | E | F | G | H | I | J | K | L | M | N | O | P |
| --- | --- | --- | --- | --- | --- | --- | --- | --- | --- | --- | --- | --- | --- | --- | --- | --- |
| **IV_E_, IV_W_, LA** | 7 | 4 | 5 | 4 | 3 | - | 5 | - | 2 | - | - | - | - | - | - | - |
| **MCR** | 5 | - | 5 | - | 5 | 5 | 1 | 2 | - | - | - | - | - | - | - | - |
| **EBO** | 4 | 3 | 1 | 3 | 1 | - | 2 | - | - | 1 | - | - | - | - | - | - |
| **OIV_N_, OIV_S_** | 1 | - | 2 | 2 | 2 | 2 | 1 | - | - | - | 3 | - | - | - | - | - |
| **MM/MTA** | 1 | 1 | 4 | 1 | 4 | - | - | - | - | - | - | - | - | - | - | - |
| **OLO_N_, OLO_S_** | 1 | 3 | 2 | - | 2 | 1 | - | - | - | - | - | - | - | - | - | - |
| **TAK** | 1 | 3 | - | 3 | - | - | - | 1 | 1 | - | - | - | - | - | - | - |
| **LBK** | 1 | 2 | 1 | 1 | 1 | 1 | - | - | - | - | - | - | - | - | - | - |
| **MFCH** | 1 | 2 | 1 | 2 | - | - | - | - | - | - | - | - | - | - | 1 | - |
| **NKO** | - | 4 | - | 5 | - | - | - | - | - | - | - | - | - | - | - | - |
| **MBJ** | - | 1 | 2 | - | 3 | 2 | - | - | - | - | - | - | - | - | - | - |
| **NGO** | 1 | - | 1 | - | - | - | - | - | 1 | 2 | - | - | - | - | - | - |
| **DJA** | 1 | - | - | - | - | - | 1 | - | - | 1 | - | - | - | - | - | - |
| **MKB** | 2 | - | 1 | - | - | - | - | - | - | - | - | - | - | - | - | - |
| **HAO** | 2 | 1 | - | - | - | - | 1 | - | - | - | - | - | - | - | - | - |
| **CPO** | 1 | - | - | - | - | - | - | - | 1 | - | - | - | - | - | - | - |
| **DIE** | - | - | - | - | - | - | - | - | - | - | - | 1 | 1 | - | - | - |
| **KOM** | 1 | - | - | - | - | - | - | - | - | 1 | - | - | - | 1 | - | - |
| **PAN** | - | - | 1 | - | - | - | - | 1 | - | - | - | - | - | - | - | - |
| **LEF** | - | 1 | - | 1 | - | - | - | 1 | - | - | - | - | - | - | - | - |
| **ODZ** | - | - | - | - | - | - | - | - | - | - | - | - | - | - | - | 1 |
| **OMA** | - | 1 | - | 1 | - | - | - | - | - | - | - | - | - | - | - | - |
| **ND/CO** | - | 1 | - | - | - | - | - | - | - | - | - | - | - | - | - | - |
| **SA** | - | - | - | - | - | - | - | 1 | - | - | - | - | - | - | - | - |
| **Total** | 30 | 27 | 26 | 23 | 21 | 11 | 11 | 6 | 5 | 5 | 3 | 1 | 1 | 1 | 1 | 1 |

Table S4. Distribution of composite *C. callipygus* haplotypes by site.

| **Site codes** | A | B | C | D | E | F | G | H | I | J | K | L | M | N | O | P | Q | R | S | T | U | V | W | X | Y | Z | Φ |
| --- | --- | --- | --- | --- | --- | --- | --- | --- | --- | --- | --- | --- | --- | --- | --- | --- | --- | --- | --- | --- | --- | --- | --- | --- | --- | --- | --- |
| **BKO** | - | - | 1 | 1 | - | - | - | - | - | - | - | - | - | - | 1 | - | - | 1 | - | - | - | - | - | 1 | - | - | - |
| **OLO_N_** | 5 | 3 | 8 | - | 4 | 3 | 2 | 1 | 3 | 1 | 3 | - | - | - | - | - | - | - | - | - | - | - | - | - | - | - | - |
| **OLO_S_** | 9 | 4 | 4 | - | 1 | - | - | 3 | 1 | 1 | 3 | 4 | 2 | 3 | - | - | - | - | - | - | - | - | - | - | - | - | - |
| **MFCH** | - | 3 | 2 | 3 | 2 | 2 | - | 2 | 1 | - | 1 | 1 | 1 | - | - | - | - | 1 | - | - | - | - | - | - | - | - | - |
| **MKB** | 1 | 6 | 6 | - | - | - | - | 2 | 1 | - | - | - | - | - | - | - | - | - | - | - | - | - | - | - | - | - | - |
| **LBK** | 4 | 5 | 1 | 6 | 5 | 4 | - | 1 | 2 | 1 | 2 | - | - | - | - | - | - | - | - | - | - | - | - | - | - | - | - |
| **NIG** | - | - | - | - | - | - | - | - | - | - | - | - | - | - | 2 | 3 | - | - | - | - | 1 | - | - | - | - | - | - |
| **LO_S_** | 22 | 35 | 31 | 23 | 28 | 31 | 26 | 15 | 11 | 14 | - | - | 2 | 1 | - | - | - | - | - | 2 | - | - | - | - | - | - | - |
| **CPO** | 6 | 3 | - | 2 | - | 3 | - | - | - | - | - | - | - | - | - | - | - | - | - | - | - | - | - | - | - | - | - |
| **OIV_S_** | 5 | 12 | 16 | 18 | 12 | 11 | - | 2 | - | 2 | - | - | - | - | - | - | - | - | - | - | - | - | - | - | - | - | - |
| **LO_N_** | 2 | 2 | - | 1 | 1 | 5 | - | - | - | - | - | - | - | - | - | - | - | - | - | - | - | - | - | - | - | - | - |
| **OIV_N_** | 9 | 5 | 3 | 2 | 2 | 1 | - | 1 | 2 | - | - | - | - | - | - | - | - | - | - | - | - | 2 | - | - | - | - | - |
| **MBJ** | 2 | 1 | 1 | - | 1 | 1 | - | - | - | 1 | - | - | - | - | - | - | - | - | - | - | - | - | - | - | - | - | - |
| **HAO** | - | 9 | 3 | 1 | 5 | 3 | 1 | - | 4 | 3 | 3 | 1 | - | - | - | - | - | - | - | - | - | - | - | - | 1 | - | - |
| **IV_W_** | 3 | 2 | 3 | 4 | 1 | 1 | 1 | 2 | 1 | - | 1 | - | - | - | - | - | - | - | - | - | - | - | - | - | - | - | - |
| **SA** | - | - | - | - | - | - | 1 | - | - | - | - | - | - | - | - | - | 3 | - | - | - | - | - | 1 | - | - | 1 | - |
| **IVE, LA** | 5 | 5 | 4 | 16 | 7 | 4 | 5 | 2 | 2 | 1 | - | - | - | - | - | - | - | - | - | - | - | - | - | - | - | - | - |
| **ND/CO** | 43 | 4 | 4 | 7 | 7 | 7 | - | 2 | 3 | 3 | - | 2 | - | - | - | - | - | - | 2 | - | - | - | - | - | - | - | - |
| **WA** | 1 | 5 | 4 | 4 | 5 | 4 | 3 | - | 1 | 1 | - | 2 | - | 1 | - | - | - | - | - | - | - | - | - | - | - | - | 1 |
| **BRA** | - | - | - | - | 1 | - | - | - | - | 1 | - | - | - | - | - | - | - | - | - | - | - | - | - | - | - | - | - |
| **EBO** | - | - | - | - | - | 1 | - | - | - | - | - | - | - | - | - | - | - | - | - | - | - | - | - | - | - | - | - |
| **GA** | 1 | 1 | 1 | - | - | 1 | 1 | 1 | - | - | - | - | - | - | - | - | - | - | - | - | - | - | 1 | - | - | - | - |
| **MCR** | - | - | - | - | - | - | - | - | - | - | - | 1 | - | - | - | - | - | - | - | - | - | - | - | - | - | - | - |
| **MTZ** | 1 | - | - | - | - | - | - | - | - | - | - | - | - | - | - | - | - | - | - | - | - | - | - | - | - | - | - |
| **MM/MTA** | - | - | - | - | - | - | - | 1 | - | - | - | - | - | - | - | - | - | - | - | - | - | - | - | - | - | - | - |
| **KOM** | - | - | - | - | - | - | - | - | - | - | - | 1 | - | - | - | - | - | - | - | - | - | - | - | - | - | - | - |
| **TAK** | - | - | - | - | 1 | - | - | - | - | - | - | - | - | - | - | - | - | - | - | - | 1 | - | - | - | - | - | - |
| **Total** | 119 | 105 | 92 | 88 | 83 | 82 | 40 | 35 | 32 | 29 | 13 | 12 | 5 | 5 | 3 | 3 | 3 | 2 | 2 | 2 | 2 | 2 | 2 | 1 | 1 | 1 | 1 |

Table S5. Distribution of composite *P. monticola* haplotypes by site.

| **Site codes** | A | B | C | D | E | F | G | H | I | J | K | L | M | N | O | P | Q | R | S | T | U | V | W | X | Y | |
| --- | --- | --- | --- | --- | --- | --- | --- | --- | --- | --- | --- | --- | --- | --- | --- | --- | --- | --- | --- | --- | --- | --- | --- | --- | --- | --- |
| **BKO** | - | - | - | - | - | - | - | 2 | - | - | - | - | - | - | - | - | - | - | - | - | - | - | - | - | - | |
| **CPO** | 11 | 14 | 9 | 4 | - | - | - | - | 1 | - | - | - | - | - | - | 4 | - | - | - | - | - | - | - | - | - | |
| **DENG** | 1 | - | - | - | - | - | - | - | 1 | - | - | - | - | - | - | - | - | - | - | - | - | - | - | 1 | - | |
| **EBO** | 4 | 1 | 1 | - | - | 1 | - | 8 | 9 | - | - | - | 5 | - | - | - | - | - | - | - | - | - | - | - | - | |
| **EDEA** | - | 1 | - | 1 | - | - | - | 4 | 4 | - | - | 7 | - | - | - | - | - | - | - | - | - | - | - | - | - | |
| **EKA** | 2 | 1 | 1 | 1 | - | - | - | - | - | - | - | - | - | - | - | - | - | - | - | - | - | - | - | - | - | |
| **GA** | 11 | 32 | 6 | - | - | 3 | - | - | - | - | - | - | - | - | - | - | - | - | - | - | - | - | - | - | - | |
| **HAO** | 2 | 1 | 1 | - | - | - | - | - | - | - | - | - | - | - | 3 | - | - | - | - | 1 | - | - | - | - | - | |
| **IV_W_, IV_E_, LA** | 19 | 12 | 15 | 7 | 1 | 1 | - | - | - | - | - | - | - | - | - | - | - | - | - | - | - | - | - | - | - | |
| **KOM** | 1 | 1 | 1 | - | - | - | - | - | - | - | - | - | 2 | - | 1 | - | - | - | - | - | - | - | - | - | 1 | |
| **LBK** | 3 | 2 | 2 | 1 | 1 | 1 | - | - | - | - | - | - | - | - | - | - | - | - | - | - | - | - | - | - | - | |
| **LO_N_, LO_S_** | 32 | 18 | 18 | 9 | 14 | 5 | 11 | - | - | - | 1 | - | - | - | - | - | - | - | - | - | - | - | - | - | - | |
| **MCR** | 11 | 3 | 14 | 2 | 7 | 4 | 2 | - | - | - | - | - | - | - | - | - | - | - | - | - | - | - | - | - | - | |
| **MFCH** | 6 | 4 | 2 | 2 | 4 | 2 | 3 | - | - | - | 1 | - | - | - | - | - | - | - | - | - | - | - | - | - | - | |
| **MKB** | 3 | 4 | 2 | 2 | 2 | 1 | - | - | - | - | - | - | - | - | - | - | - | - | - | - | - | - | - | - | - | |
| **MM/MTA** | 26 | 25 | 21 | 19 | 2 | 5 | 4 | - | - | - | 6 | - | 2 | - | - | - | - | - | - | - | - | 1 | - | - | - |  |
| **MOO_N_, MOO_S_** | 4 | 2 | 1 | 2 | 1 | - | - | - | - | - | - | - | - | - | - | - | - | - | - | - | - | - | - | - | - |  |
| **ND/CO** | 26 | 7 | 12 | 1 | 5 | 3 | 3 | - | - | - | 3 | - | - | - | - | - | - | - | - | - | - | - | - | - | - |  |
| **NKO** | 1 | 3 | 1 | - | 1 | 1 | - | 1 | - | - | - | - | 1 | - | - | - | - | - | - | - | - | - | - | - | - |  |
| **NIG** | - | - | - | - | - | - | - | 2 | 1 | 24 | - | - | - | 7 | - | - | - | 2 | - | - | - | - | - | - | - |  |
| **OIV_N_, OIV_S_** | 4 | 5 | 3 | 2 | 3 | 3 | 3 | 1 | 1 | - | 3 | - | - | - | - | - | - | - | - | - | - | - | - | - | - |  |
| **OLO_N_, OLO_S_** | 9 | 4 | 9 | 3 | 6 | 5 | 3 | - | - | - | 7 | - | - | - | - | - | - | - | - | - | - | - | - | - | - |  |
| **PAN** | 1 | - | - | - | 1 | - | - | - | - | - | - | - | - | - | - | - | - | - | - | - | - | - | - | - | - |  |
| **TAK** | 1 | - | - | - | - | - | - | 7 | 6 | - | - | 6 | 2 | - | - | - | - | - | - | - | - | - | - | - | - |  |
| **DIV** | - | - | - | - | - | - | - | - | - | - | - | - | - | - | - | - | - | - | 1 | - | - | - | - | - | - |  |
| **CAP** | - | - | - | - | - | - | - | - | - | - | - | - | - | - | - | - | - | - | - | - | 1 | - | - | - | - |  |
| **KIS** | - | - | - | - | - | - | - | - | - | - | - | - | - | - | - | - | 1 | - | - | - | - | - | - | - | - |  |
| **LEF** | - | - | - | - | - | - | - | - | - | - | - | - | - | - | - | - | - | - | - | - | - | - | 1 | - | - |  |
| **OMA** | - | - | - | - | - | - | - | 1 | 1 | - | - | - | - | - | - | - | - | - | - | - | - | - | - | - | - |  |
| **SA** | - | - | - | - | - | - | - | - | - | - | - | - | - | - | - | - | 1 | - | - | - | - | - | - | - | - |  |
| **WA** | 1 | - | 1 | - | 1 | 1 | 1 | - | - | - | - | - | - | - | - | - | - | - | - | - | - | - | - | - | - |  |
| **Total** | 179 | 140 | 120 | 56 | 49 | 36 | 30 | 26 | 24 | 24 | 21 | 13 | 12 | 7 | 4 | 4 | 2 | 2 | 1 | 1 | 1 | 1 | 1 | 1 | 1 |  |

Table S6. Mitochondrial diversity indices for each species by site.

|  | ***C. dorsalis*** | | | | ***C. callipygus*** | | | | ***P. monticola*** | | | |
| --- | --- | --- | --- | --- | --- | --- | --- | --- | --- | --- | --- | --- |
| **Site codes** | **n** | **Hd** | **π** | **θ** | **n** | **Hd** | **π** | **θ** | **n** | **Hd** | **π** | **θ** |
| BKO |  |  |  |  | 5 | 1 | 0.079 | 0.073 |  |  |  |  |
| CPO |  |  |  |  | 12 | 0.939 | 0.053 | 0.047 | 21 | 0.986 | 0.066 | 0.080 |
| EBO | 9 | 0.944 | 0.039 | 0.0402 |  |  |  |  | 19 | 0.942 | 0.076 | 0.059 |
| EDEA |  |  |  |  |  |  |  |  | 8 | 0.964 | 0.045 | 0.054 |
| GA |  |  |  |  |  |  |  |  | 33 | 0.769 | 0.034 | 0.031 |
| HAO |  |  |  |  | 15 | 0.952 | 0.063 | 0.066 | 7 | 0.857 | 0.038 | 0.043 |
| IV_E_ | 5 | 1 | 0.048 | 0.045 | 17 | 0.985 | 0.060 | 0.058 | 9 | 1 | 0.041 | 0.045 |
| IV_W_ |  |  |  |  | 12 | 0.939 | 0.071 | 0.064 | 7 | 1 | 0.052 | 0.051 |
| KOM |  |  |  |  |  |  |  |  | 5 | 0.9 | 0.053 | 0.047 |
| LA | 9 | 0.972 | 0.038 | 0.036 | 21 | 0.957 | 0.055 | 0.047 | 6 | 0.8 | 0.040 | 0.041 |
| LBK | 5 | 1 | 0.056 | 0.054 | 16 | 0.983 | 0.067 | 0.070 | 4 | 1 | 0.046 | 0.047 |
| LO_N_ |  |  |  |  | 8 | 0.821 | 0.044 | 0.038 | 7 | 0.857 | 0.038 | 0.037 |
| LO_S_ |  |  |  |  | 135 | 0.981 | 0.074 | 0.062 | 33 | 0.981 | 0.043 | 0.052 |
| MBJ | 4 | 1 | 0.042 | 0.045 | 5 | 0.9 | 0.066 | 0.063 |  |  |  |  |
| MCR | 12 | 0.788 | 0.043 | 0.033 |  |  |  |  | 16 | 0.950 | 0.057 | 0.055 |
| MFCH | 5 | 0.9 | 0.048 | 0.047 | 8 | 0.929 | 0.071 | 0.069 | 9 | 0.972 | 0.054 | 0.057 |
| MKB |  |  |  |  | 11 | 0.945 | 0.046 | 0.048 | 4 | 0.833 | 0.037 | 0.038 |
| MM/MTA | 6 | 0.933 | 0.039 | 0.037 |  |  |  |  | 35 | 0.96 | 0.047 | 0.054 |
| MOO_S_ |  |  |  |  |  |  |  |  | 5 | 0.700 | 0.042 | 0.043 |
| ND/CO |  |  |  |  | 60 | 0.972 | 0.048 | 0.049 | 30 | 0.993 | 0.052 | 0.063 |
| NGO | 4 | 1 | 0.045 | 0.046 |  |  |  |  |  |  |  |  |
| NIG |  |  |  |  | 6 | 0.733 | 0.054 | 0.046 | 35 | 0.931 | 0.073 | 0.074 |
| NKO | 5 | 0.9 | 0.003 | 0.003 |  |  |  |  | 6 | 0.933 | 0.069 | 0.073 |
| OIV_N_ | 6 | 0.933 | 0.050 | 0.044 | 20 | 0.942 | 0.067 | 0.058 | 4 | 0.833 | 0.042 | 0.041 |
| OIV_S_ |  |  |  |  | 34 | 0.918 | 0.054 | 0.050 |  |  |  |  |
| OLO_N_ | 4 | 0.5 | 0.024 | 0.027 | 23 | 0.933 | 0.060 | 0.055 |  |  |  |  |
| OLO_S_ |  |  |  |  | 23 | 0.953 | 0.066 | 0.061 | 9 | 0.917 | 0.037 | 0.039 |
| SA |  |  |  |  | 5 | 1 | 0.067 | 0.069 |  |  |  |  |
| TAK | 6 | 1 | 0.037 | 0.037 |  |  |  |  | 10 | 0.711 | 0.058 | 0.054 |
| WA |  |  |  |  | 11 | 0.982 | 0.066 | 0.067 |  |  |  |  |

Table S7. *C. dorsalis* expected heterozygosity and tests of deviation from Hardy-Weinberg Equilibrium.

|  |  | **He** | | **Hardy-Weinberg Equilibrium test** | | | | | | | | | | |
| --- | --- | --- | --- | --- | --- | --- | --- | --- | --- | --- | --- | --- | --- | --- |
|  | **n** | **Mean** | **SD** | **BM2113** | **INRA40** | **BM1225** | **BM143** | **INRA05** | **BM1862** | **BM864** | **MM12** | **BM121** | **BM848** | **SR12** |
| EBO | 6 | 0.769 | 0.121 | 0.066 | 0.860 | 0.284 | NT | NT | NT | NT | 1 | 0.060 | 0.772 | 0.393 |
| IVE | 4 | 0.724 | 0.231 | 1 | 1 | 0.658 | NT | NT | NT | NT | 0.198 | 1 | 0.197 | 1 |
| LA | 5 | 0.737 | 0.161 | 1 | 0.232 | 0.691 | NT | NT | NT | NT | 0.430 | 0.466 | 0.601 | 0.428 |
| MM/MTA | 5 | 0.647 | 0.259 | 1 | 1 | 0.657 | NT | NT | NT | NT | 1 | 0.028 | 0.622 | 1 |
| OIV_N_ | 5 | 0.717 | 0.171 | 0.334 | 1 | 0.317 | 0.851 | 1 | 1 | 0.600 | 1 | 0.191 | 0.849 | 1 |
| OLO_S_ | 4 | 0.769 | 0.220 | 1 | 1 | NT | 1 | 0.334 | 1 | 1 | 0.471 | 1 | 0.467 | 0.197 |

NT: Not tested because of lack of polymorphism and/or missing data.

Table S8. *C. callipygus* expected heterozygosity and tests of deviation from Hardy-Weinberg Equilibrium.

|  |  | **He** | | **Hardy-Weinberg Equilibrium test** | | | | | | | | | | |
| --- | --- | --- | --- | --- | --- | --- | --- | --- | --- | --- | --- | --- | --- | --- |
|  | **n** | **Mean** | **SD** | **BM2113** | **INRA40** | **BM1225** | **MM12** | **BM121** | **BM848** | **SR12** | **BM143** | **INRA05** | **BM1862** | **BM864** |
| HAO | 6 | 0.616 | 0.243 | 1 | 0.713 | 0.048* | 1 | 1 | 1 | 0.110 | NT | 1 | 0.089 | 0.200 |
| IV_E_ | 11 | 0.697 | 0.209 | 0.016* | 1 | 0.108 | 0.198 | 1 | 0.763 | 0.003 | NT | NT | NT | NT |
| IV_W_ | 7 | 0.741 | 0.189 | 0.009** | 0.164 | 0.077 | 0.274 | 0.130 | 0.104 | 0.021 | NT | NT | NT | NT |
| LA | 5 | 0.533 | 0.297 | 0.127 | 0.314 | 1 | 1 | 0.047 | 1 | 0.084 | NT | NT | NT | NT |
| LBK | 11 | 0.583 | 0.258 | 0.017* | 1 | 0.406 | 1 | 0.277 | 0.725 | 0.009 | NT | NT | NT | NT |
| LO_N_ | 6 | 0.605 | 0.195 | 0.430 | 0.311 | 1 | 1 | 0.400 | 1 | 0.047 | 0.066 | 1 | 0.427 | NT |
| LO_S_ | 79 | 0.651 | 0.247 | 0.901 | 0.017* | 0.206 | 0.049 | 0.069 | 0.538 | <0.001*** | 0.073 | 0.889 | 1 | <0.001*** |
| MFCH | 4 | 0.693 | 0.275 | 0.303 | 0.655 | 0.312 | NT | 0.428 | 1 | NT | NT | NT | NT | NT |
| MKB | 7 | 0.623 | 0.318 | 0.496 | 1 | 0.254 | 1 | 0.077 | 0.160 | 0.003** | NT | NT | NT | NT |
| ND/CO | 14 | 0.597 | 0.269 | 0.274 | 0.414 | 0.254 | 1 | 0.436 | 0.355 | 0.002** | NT | NT | NT | NT |
| OIV_N_ | 12 | 0.638 | 0.231 | 0.109 | 0.132 | 0.864 | 1 | 0.343 | 0.244 | <0.001*** | 0.332 | 1 | 1 | 0.067 |
| OIV_S_ | 17 | 0.560 | 0.287 | 0.530 | 0.959 | 0.009 | 1 | 0.738 | 0.810 | <0.001*** | 0.197 | 1 | 1 | 1 |
| OLO_N_ | 18 | 0.629 | 0.186 | 0.878 | 0.982 | 0.821 | 1 | 0.104 | 0.090 | <0.001*** | 1 | 0.516 | NT | 0.510 |
| OLO_S_ | 18 | 0.569 | 0.262 | 0.997 | 0.008** | 0.223 | 1 | 0.378 | 0.611 | <0.001*** | 0.085 | 0.194 | 1 | 0.015* |
| WA | 4 | 0.663 | 0.185 | 0.658 | 1 | 1 | 1 | 1 | 1 | 0.200 | NT | NT | NT | NT |

Asterisk indicates significance levels: *significant (P < 0.05); **very significant (P< 0.01); ***highly significant (P < 0.001).

NT: Not tested because of non-polymorphism or missing data.

Table S9. *P. monticola* expected heterozygosity and tests of deviation from Hardy-Weinberg Equilibrium.

|  |  | **He** | | **Hardy-Weinberg Equilibrium test** | | | | | | | | | | | |
| --- | --- | --- | --- | --- | --- | --- | --- | --- | --- | --- | --- | --- | --- | --- | --- |
|  | **n** | **Mean** | **SD** | **BM2113** | **INRA40** | **BM1225** | **MM12** | **BM121** | **BM848** | **SR12** | **BM143** | **INRA05** | **BM1862** | **BM864** |  |
| CPO | 18 | 0.788 | 0.124 | 0.850 | 0.708 | 0.787 | **0.004** | 0.388 | 0.294 | 0.007 | NT | NT | 1 | NT |  |
| DENG | 5 | 0.849 | 0.080 | 1 | 1 | 1 | 1 | 1 | 0.659 | 1 | NT | NT | 1 | NT |  |
| EBO | 10 | 0.872 | 0.065 | 0.147 | 0.338 | 0.335 | 0.661 | 0.200 | 0.039* | 0.283 | NT | NT | NT | NT |  |
| EDEA | 8 | 0.781 | 0.129 | 1 | 0.910 | 0.054 | 0.025 | 0.657 | 1 | 1 | NT | NT | 0.270 | NT |  |
| GA | 6 | 0.767 | 0.109 | 0.600 | 0.658 | 0.584 | 0.254 | 0.071 | 0.655 | 0.358 | NT | NT | NT | NT |  |
| HAO | 4 | 0.797 | 0.173 | 0.464 | 1 | 0.425 | 1 | 0.199 | 0.313 | 0.127 | 1 | NT: | 1 | NT: |  |
| IV_E_ | 7 | 0.849 | 0.149 | 1 | 0.506 | 1 | 1 | 0.127 | 0.144 | 0.114 | NT | NT | NT | NT |  |
| IV_W_ | 7 | 0.789 | 0.177 | 1 | 0.398 | 0.165 | 0.706 | 0.010* | 0.055 | 0.400 | NT | NT | NT | NT |  |
| LA | 5 | 0.822 | 0.062 | 1 | 1 | 1 | 1 | 0.085 | 0.237 | 1 | NT | NT | NT | NT |  |
| LO_N_ | 5 | 0.670 | 0.148 | 0.692 | 0.186 | 1 | 1 | 0.201 | 1 | 1 | NT | NT | 1 | NT |  |
| LO_S_ | 28 | 0.780 | 0.137 | 0.488 | 0.222 | 0.246 | 1 | <0.001*** | 0.295 | 0.130 | NT | 0.333 | 0.936 | 0.085 |  |
| MCR | 8 | 0.826 | 0.126 | 1 | 0.134 | 0.782 | 0.491 | 0.203 | 0.051 | 0.046 | NT | NT | NT | NT |  |
| MFCH | 9 | 0.779 | 0.131 | 0.016* | 0.038 | 0.225 | 0.496 | 0.703 | 0.575 | 0.030 | NT | NT | NT | NT |  |
| MM/MTA | 17 | 0.840 | 0.085 | 0.985 | 0.774 | 0.738 | 0.172 | 0.045* | 0.4959 | 0.046 | NT | NT | NT | NT |  |
| MOO_N_/  MOO_S_ | 6 | 0.802 | 0.162 | 0.392 | 0.614 | 0.619 | 0.393 | 0.468 | 0.0607 | 0.088 | NT | 1 | 0.194 | 1 |  |
| ND/CO | 17 | 0.778 | 0.148 | 0.064 | 0.013 | 0.123 | 0.267 | 0.008 | 0.020 | 0.106 | NT | NT | NT | NT |  |
| NGO | 55 | 0.817 | 0.108 | 0.012 | 0.209 | 0.077 | 0.119 | <0.001*** | 0.004 | 0.001 | 1 | 0.335 | 0.831 | 0.048 |  |
| NIG | 6 | 0.844 | 0.137 | 1 | 0.031 | 1 | 0.636 | 0.120 | 1 | 1 | NT | NT | NT | NT |  |
| NKO | 5 | 0.818 | 0.172 | 0.017 | 1 | 0.850 | 1 | 1 | 1 | 0.466 | NT | NT | 1 | NT |  |
| OIV_N_ | 4 | 0.706 | 0.186 | 0.313 | 0.773 | 0.317 | 1 | 1 | 0.464 | 1 | NT: | 1 | 1 | 0.202 |  |
| OLO_S_ | 6 | 0.749 | 0.223 | 0.831 | 0.287 | 0.419 | 0.624 | 0.010 | 0.300 | 0.077 | 1 | NT | 1 | 1 |  |
| TAK | 6 | 0.758 | 0.234 | 0.713 | 0.169 | 1 | 1 | 0.027 | 0.154 | 0.333 | NT | NT | NT | NT |  |

Asterisk indicates significance levels: *significant (P < 0.05); **very significant (P< 0.01); ***highly significant (P < 0.001).

NT: Not tested because of non-polymorphism or missing data.

Table S10. Significant associations between pairs of loci for *C. callipygus* and *P. monticola*

| *C. callipygus* | | | | | | | | | | | | | |
| --- | --- | --- | --- | --- | --- | --- | --- | --- | --- | --- | --- | --- | --- |
|  | **n** | **BM2113** | **INRA40** | **BM1225** | **MM12** | **BM121** | **BM848** | **SR12** | **BM143** | **INRA05** | **BM1862** | **BM864** | **P-value** |
| IV_W_ | 7 | X | X |  |  |  |  |  |  |  |  |  | <0.001*** |
| LO_N_ | 6 |  |  |  |  |  |  | X | X |  |  |  | <0.001*** |
| OIV_N_ | 12 |  |  |  |  |  |  | X |  | X |  |  | <0.001*** |
| OLO_N_ | 18 |  |  |  |  | X |  |  |  | X |  |  | <0.001*** |
| OLO_S_ | 18 |  | X |  |  |  |  | X |  |  |  |  | <0.001*** |
| *P. monticola* | | | | | | | | | | | | | |
|  | **n** | **BM2113** | **INRA40** | **BM1225** | **MM12** | **BM121** | **BM848** | **SR12** | **BM143** | **INRA05** | **BM1862** | **BM864** | **P-value** |
| EBO | 10 | X | X |  |  |  |  |  |  |  |  |  | <0.001*** |
| LO_S_ | 28 | X | X |  |  |  |  |  |  |  |  |  | <0.001*** |
|  |  | X |  | X |  |  |  |  |  |  |  |  | <0.001*** |

Asterisk indicates significance levels: *significant (P < 0.05); **very significant (P< 0.01); ***highly significant (P < 0.001).

“X” indicates where there was significant Linkage Disequilibrium.

Table S11. Probability of identity (PI_sibs_) for each locus (within species) and cumulative probabilities of identity across multiple loci for each species.

|  | *C. dorsalis* | | *C. callipygus* | | *P. monticola* | |
| --- | --- | --- | --- | --- | --- | --- |
| Loci | Per locus PI_sibs_ | Cumulative PI_sibs_ | Per locus PI_sibs_ | Cumulative PI_sibs_ | Per locus PI_sibs_ | Cumulative PI_sibs_ |
| BM121 | 0.331 | 0.330 | 0.518 | 0.518 | 0.309 | 0.309 |
| BM1225 | 0.370 | 0.122 | 0.368 | 0.190 | 0.351 | 0.109 |
| BM143 | 0.336 | 0.041* | 0.327 | 0.062 | 0.320 | 0.035* |
| BM1862 | 0.542 | 0.022* | 0.845 | 0.053 | 0.506 | 0.018* |
| BM2113 | 0.530 | 0.012* | 0.328 | 0.017* | 0.296 | 0.005** |
| BM848 | 0.323 | 0.004** | 0.548 | 0.009** | 0.369 | 0.002** |
| BM864 | 0.425 | 0.002** | 0.386 | 0.004** | 0.336 | <0.001*** |
| INRA05 | 0.577 | <0.001*** | 0.493 | 0.002** | 0.434 | <0.001*** |
| INRA40 | 0.315 | <0.001*** | 0.279 | 0.001** | 0.289 | <0.001*** |
| MM12 | 0.465 | <0.001*** | 0.705 | <0.001*** | 0.473 | <0.001*** |
| SR12 | 0.573 | <0.001*** | 0.521 | <0.001*** | 0.310 | <0.001*** |

Asterisk indicates significance levels: *significant (P < 0.05); **very significant (P< 0.01); ***highly significant (P < 0.001).

Table S12. The among group component of the total variance (F_CT_) for hypothesized *C. dorsalis* groupings.

| **Models** | **Number of groups** | **F_CT_** | **P-value** |
| --- | --- | --- | --- |
| Gulf of Guinea refugium (TAK) versus all other sites | 2 | -0.026 | 0.670 |
| Central African refugia (TAK) + (MM/MTA, MCR) + (MFCH) + (all other sites) | 4 | 0.027 | 0.239 |
| Sanaga fluvial refugium (MBJ, EBO, NKO) versus all other sites | 2 | 0.000 | 0.341 |
| Ogooué River barrier: Ogooué north (IV_W_, LA, OIV_N_) versus Ogooué south (OLO_S_) | 2 | 0.145 | 0.235 |
| Sanaga River barrier: Sanaga north (EBO, MBJ) versus Sanaga south (NKO) | 2 | -0.026 | 0.671 |
| Sanaga and Ogooué River barriers: Sanaga north (EBO, MBJ) versus Sanaga south and Ogooué North (NKO, MM/MTA, MCR, OIV_N_, IV_W_, LA, LBK, NGO) versus Ogooué south (OLO_S_, MFCH) | 3 | 0.008 | 0.274 |

Table S13. The among group component of the total variance (F_CT_) for hypothesized *C. callipygus* groupings.

| **Models** | **Number of groups** | **F_CT_** | **P-value** |
| --- | --- | --- | --- |
| Gulf of Guinea refugium (NIG/TAK) versus all other sites | 2 | 0.195 | 0.0498* |
| Central African refugia (NIG/TAK) + (CPO) + (MFCH) versus all other sites | 4 | 0.055 | 0.136 |
| Sanaga fluvial refugium (CPO, MBJ) versus all other sites | 2 | -0.012 | 0.534 |
| Ogooué River barrier: Ogooué north (LO_N_, OIV_N_, LA, OLO_N_, HAO) versus Ogooué south (LO_S_, OIV_S_, OLO_S_) | 2 | -0.012 | 0.795 |
| Sanaga River barrier: | NT | NT | NT |
| Sanaga and Ogooué River barriers: Sanaga north (NIG/TAK, EBO, MBJ, LIN) versus Sanaga south and Ogooué North (CPO, MKB, ND/CO, LBK, LO_N_, OIV_N_, OLO_N_, IV_W_, IV_E_, LA, HAO, ,) versus Ogooué south (LO_S_, OIV_S_, OLO_S_, MFCH, WA). | 3 | -0.003 | 0.399 |

Asterisk indicates significance levels: *significant (P < 0.05)

Table S14. Among group component of the total variance (F_CT_) for hypothesized *P. monticola* groupings.

| **Models** | **Number of groups** | **F_CT_** | **P-value** |
| --- | --- | --- | --- |
| Gulf of Guinea refugium (NIG/TAK/BAM) versus all other sites | 2 | 0.283 | 0.068‡ |
| Central African upland refugia (NIG/TAK/BAM) + (CPO) + (MM/MTA, MCR) + (MCFH) versus all other sites | 5 | -0.008 | 0.458 |
| Sanaga fluvial refugium (CPO, EDEA, EBO, KOM, DENG, NKO, EKA) versus all other sites | 2 | 0.064 | 0.019* |
| Ogooué River barrier: Ogooué north (LO_N_, OIV_N_, LA, HAO, IV_W_, IW_E_) versus Ogooué south (MOO_S_, LO_S_, OIV_S_, OLO_S_) | 2 | -0.034 | 0.777 |
| Sanaga River barrier: Sanaga north (EBO) versus Sanaga south (CPO, EDEA, KOM, DENG, NKO, EKA) | 2 | -0.103 | 0.720 |
| Sanaga and Ogooué River barriers: Sanaga north (NIG/TAK/BAM, EBO) versus Sanaga south and Ogooué north (CPO, EDEA, KOM, DENG, NKO, EKA, MM/MTA, MCR, MKB, LO_N_, OIV_N_, IV_W_, IV_E_, LA, HAO, ND/CO, LBK, NGO) versus Ogooué south (MOO_S_, LO_S_, OIV_S_, OLO_S_, MFCH, GA) | 3 | 0.100 | 0.024* |

Asterisk indicates significance levels: ‡marginally significant (P<0.10); *significant (P < 0.05)
