# Supplementary material for: Evaluating the role of Pleistocene refugia, rivers and environmental variation in the diversification of central African duikers (genera Cephalophus and Philantomba)
Source: BMC Evol Biol. 2017 Sep 6;17:212. doi: 10.1186/s12862-017-1054-4 (PMC5585889; doi:10.1186/s12862-017-1054-4)
Supplement: Supplementary file 2 — Supplementary figures. Figure S1. Graphical representation of the STRUCTURE output for the identification of unknown NGO samples for K = 2. Figure S2a. minimum Spanning Network (MSN) of C. dorsalis based on 16 composite haplotypes (collapsed haplotypes). Red circles indicate the median vector or hypothesized (ancestral) haplotypes and the length of each branch connecting sampled haplotypes indicates the genetic distance between haplotypes. b. Minimum Spanning Network (MSN) of C. callipygus based on 27 composite haplotypes (collapsed haplotypes). C. minimum Spanning Network (MSN) of P. monticola based on 25 composite haplotypes (collapsed haplotypes). Figure S3. Cytochrome c oxidase subunit I neighbour-joining bootstrap consensus phylogeny based on Tamura-Nei distances. Bootstrap values of 75% or greater are indicated at the relevant node. Figure S4a-b. Graphical representation of the STRUCTURE output for C. dorsalis for K = 2. Figure S5a-b. Graphical representation of the STRUCTURE output for C. callipygus for K = 6. Figure S6a-b. Graphical representation of the STRUCTURE output for P. monticola for K = 2. Figure S7. Environmental variables used as predictors in MAXENT and GDM analyses. Figure S8. Jackknife of regularized training gain for C. dorsalis. Fig. S9. Jackknife of regularized training gain for C. callipygus. Figure S10. Jackknife of regularized training gain for P. monticola. Figure S11. MAXENT predicted species distributions for: a) C. dorsalis b) C.callipygus and c) P. monticola. Figure S12. GDM functional response curves for C. callipygus. Figure S13. GDM functional response curves for P. monticola (PPTX 2695 kb) [file 12862_2017_1054_MOESM2_ESM.pptx]

## Slide 1
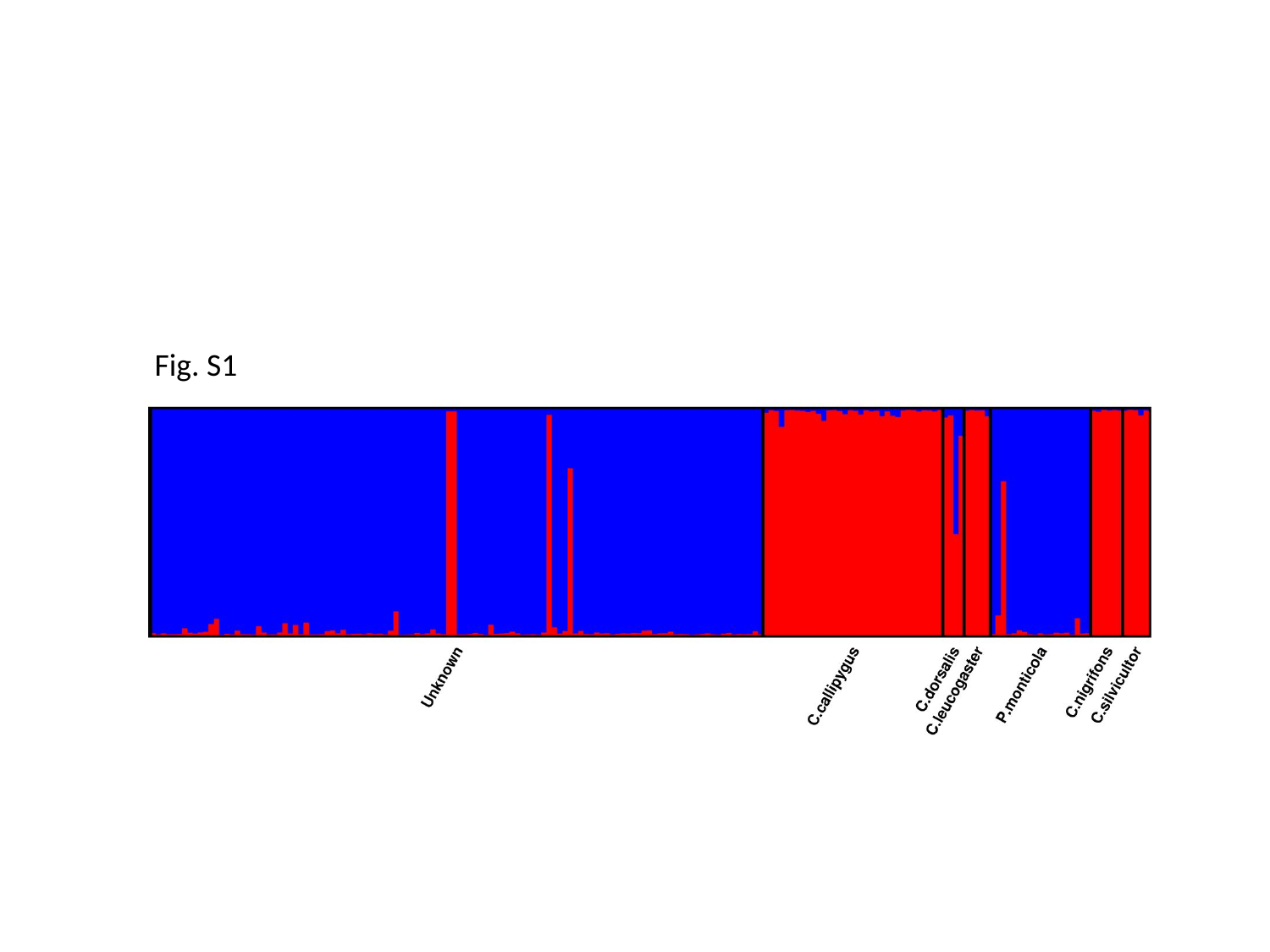

Fig. S1

## Slide 2
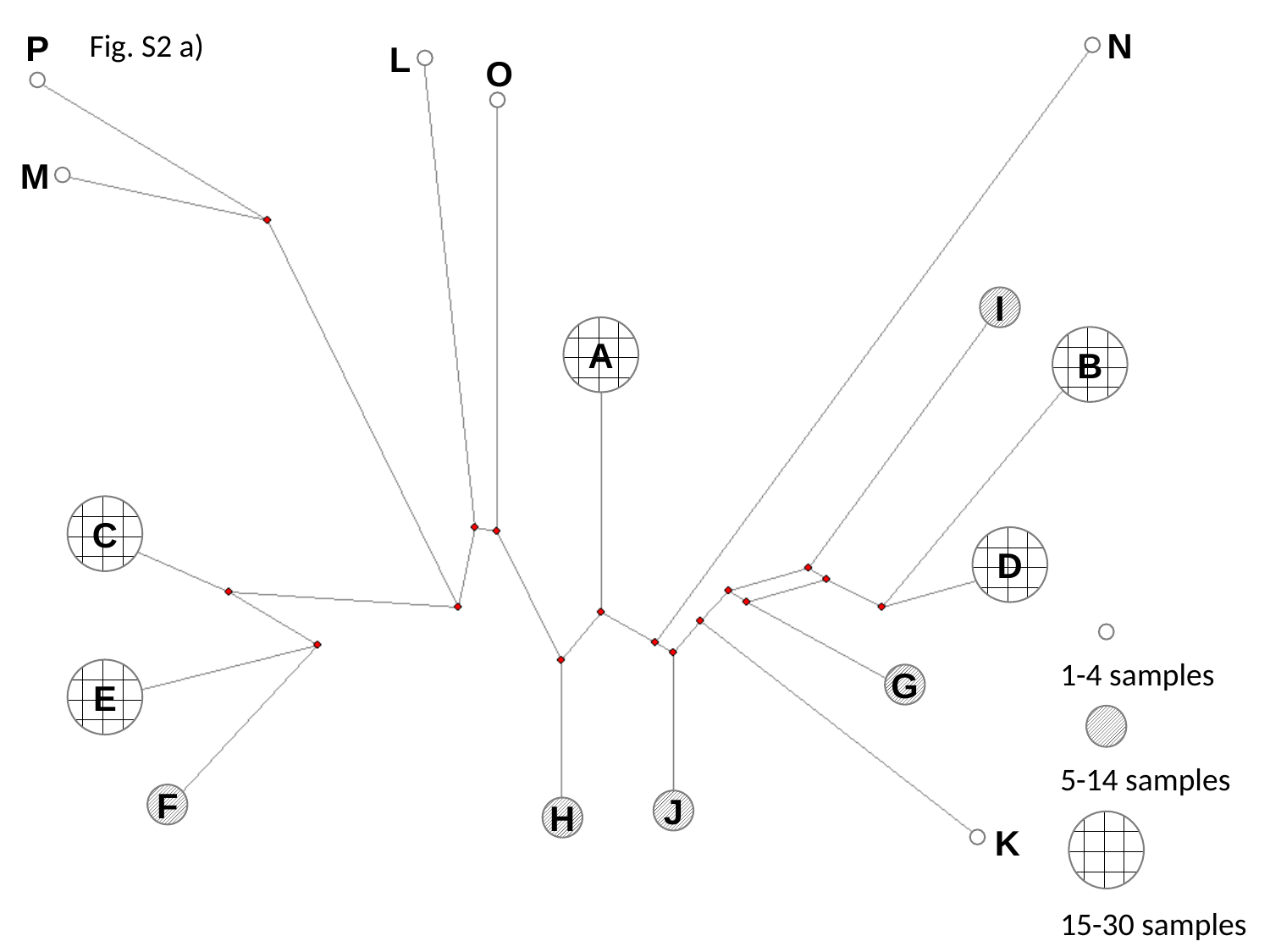

N
P
L
O
M
I
A
B
C
D
E
G
F
J
H
K
1-4 samples
5-14 samples
15-30 samples
Fig. S2 a)

## Slide 3
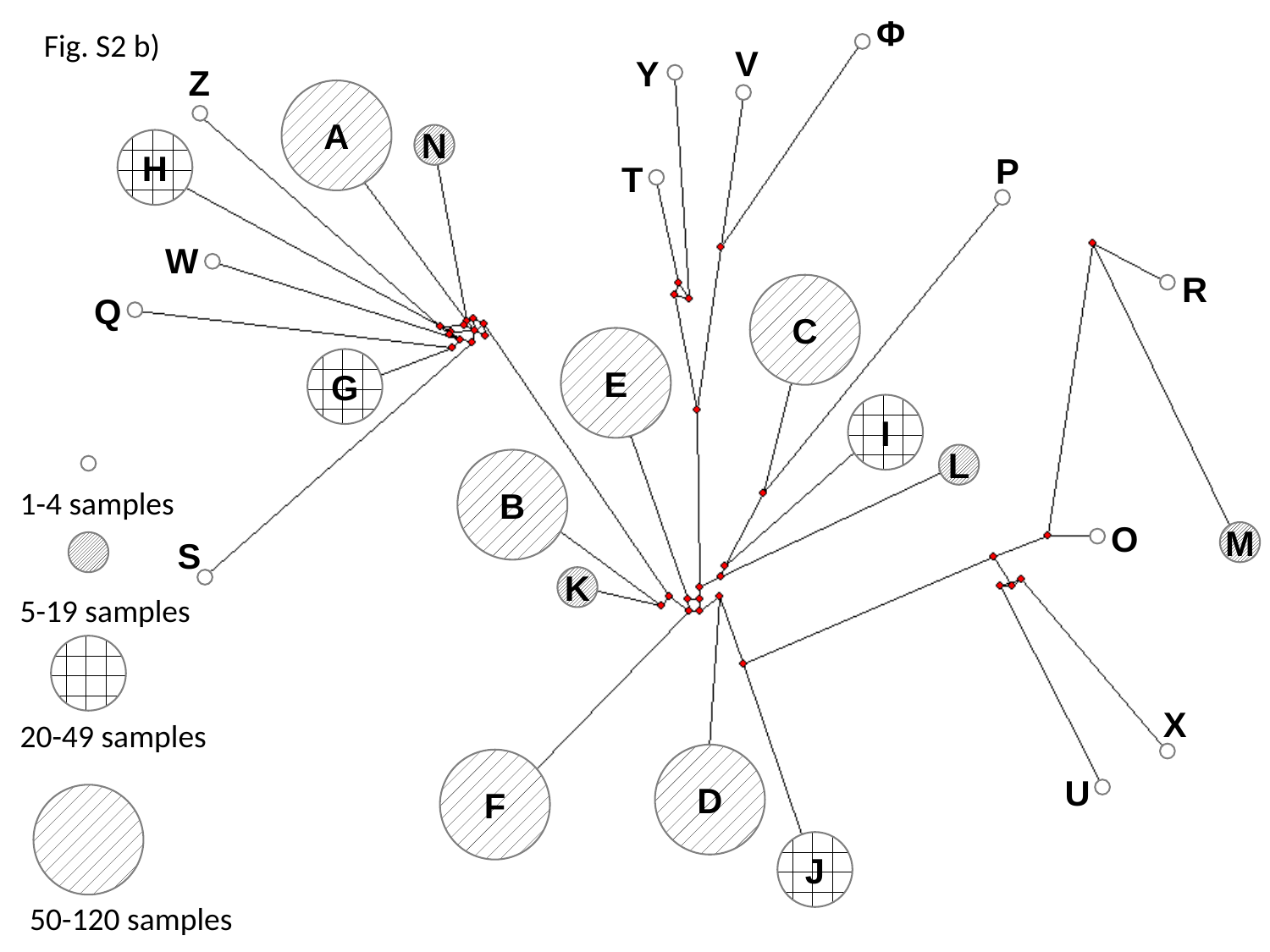

Φ
V
Y
Z
A
N
H
P
T
W
R
C
Q
E
G
I
L
B
1-4 samples
O
M
S
K
5-19 samples
X
20-49 samples
D
F
U
J
50-120 samples
Fig. S2 b)

## Slide 4
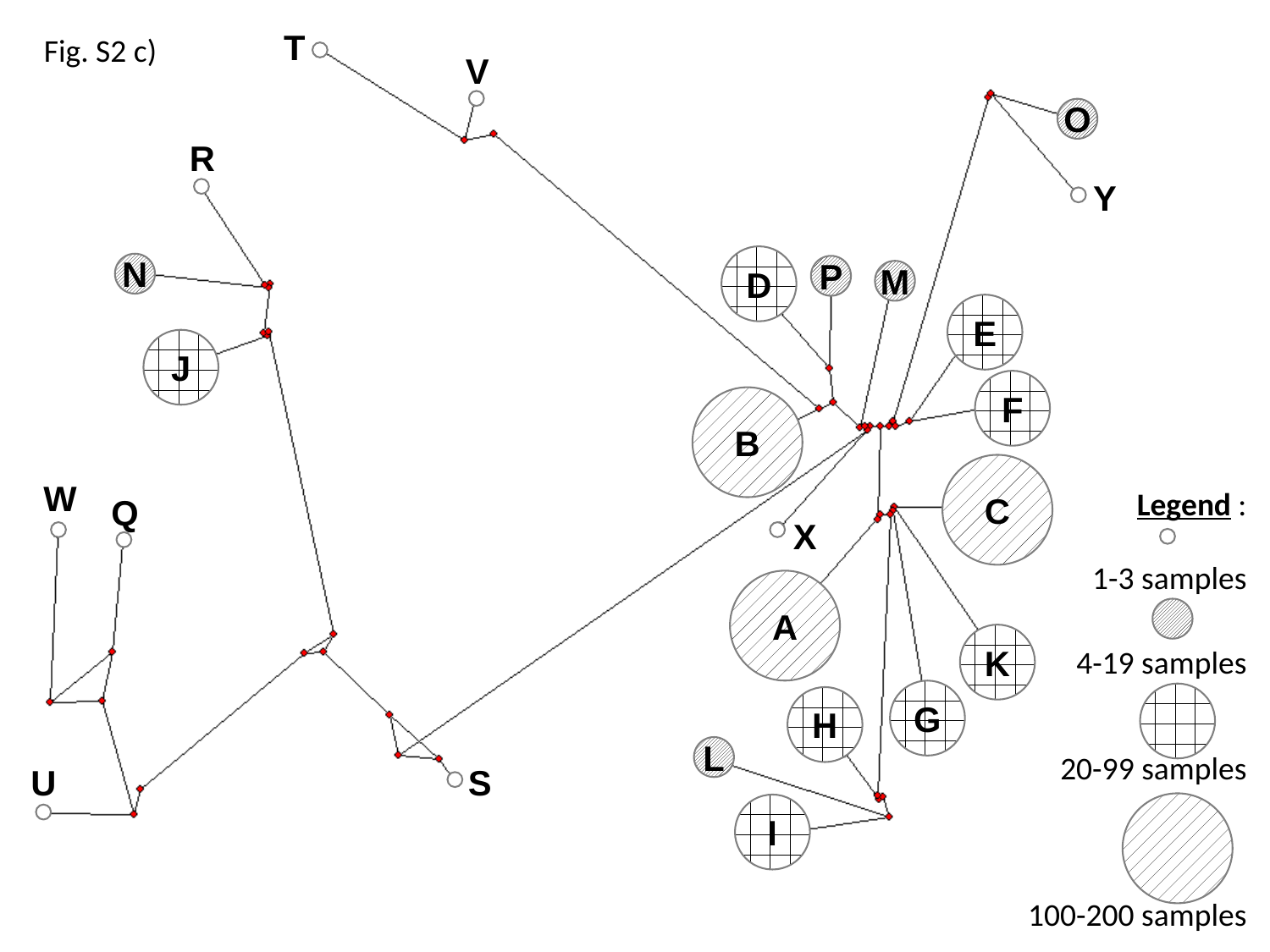

T
V
O
R
Y
D
N
P
M
E
J
F
B
C
W
Legend :
Q
X
1-3 samples
A
K
4-19 samples
G
H
L
20-99 samples
U
S
I
100-200 samples
Fig. S2 c)

## Slide 5
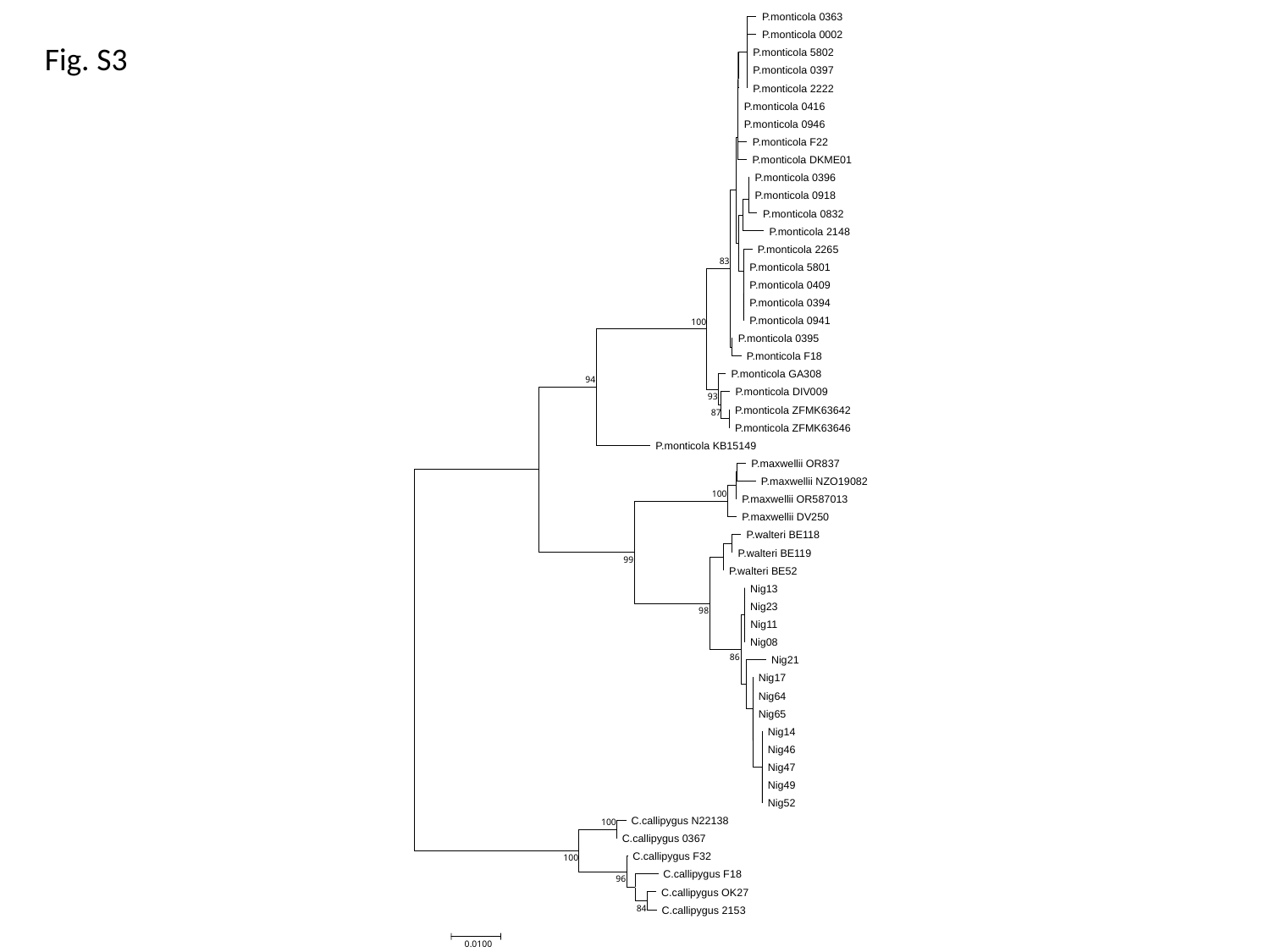

P.monticola 0363
 P.monticola 0002
 P.monticola 5802
 P.monticola 0397
 P.monticola 2222
 P.monticola 0416
 P.monticola 0946
 P.monticola F22
 P.monticola DKME01
 P.monticola 0396
 P.monticola 0918
 P.monticola 0832
 P.monticola 2148
 P.monticola 2265
83
 P.monticola 5801
 P.monticola 0409
 P.monticola 0394
 P.monticola 0941
100
 P.monticola 0395
 P.monticola F18
 P.monticola GA308
94
 P.monticola DIV009
93
 P.monticola ZFMK63642
87
 P.monticola ZFMK63646
 P.monticola KB15149
 P.maxwellii OR837
 P.maxwellii NZO19082
100
 P.maxwellii OR587013
 P.maxwellii DV250
 P.walteri BE118
 P.walteri BE119
99
 P.walteri BE52
 Nig13
 Nig23
98
 Nig11
 Nig08
86
 Nig21
 Nig17
 Nig64
 Nig65
 Nig14
 Nig46
 Nig47
 Nig49
 Nig52
 C.callipygus N22138
100
 C.callipygus 0367
 C.callipygus F32
100
 C.callipygus F18
96
 C.callipygus OK27
84
 C.callipygus 2153
0.0100
Fig. S3

## Slide 6
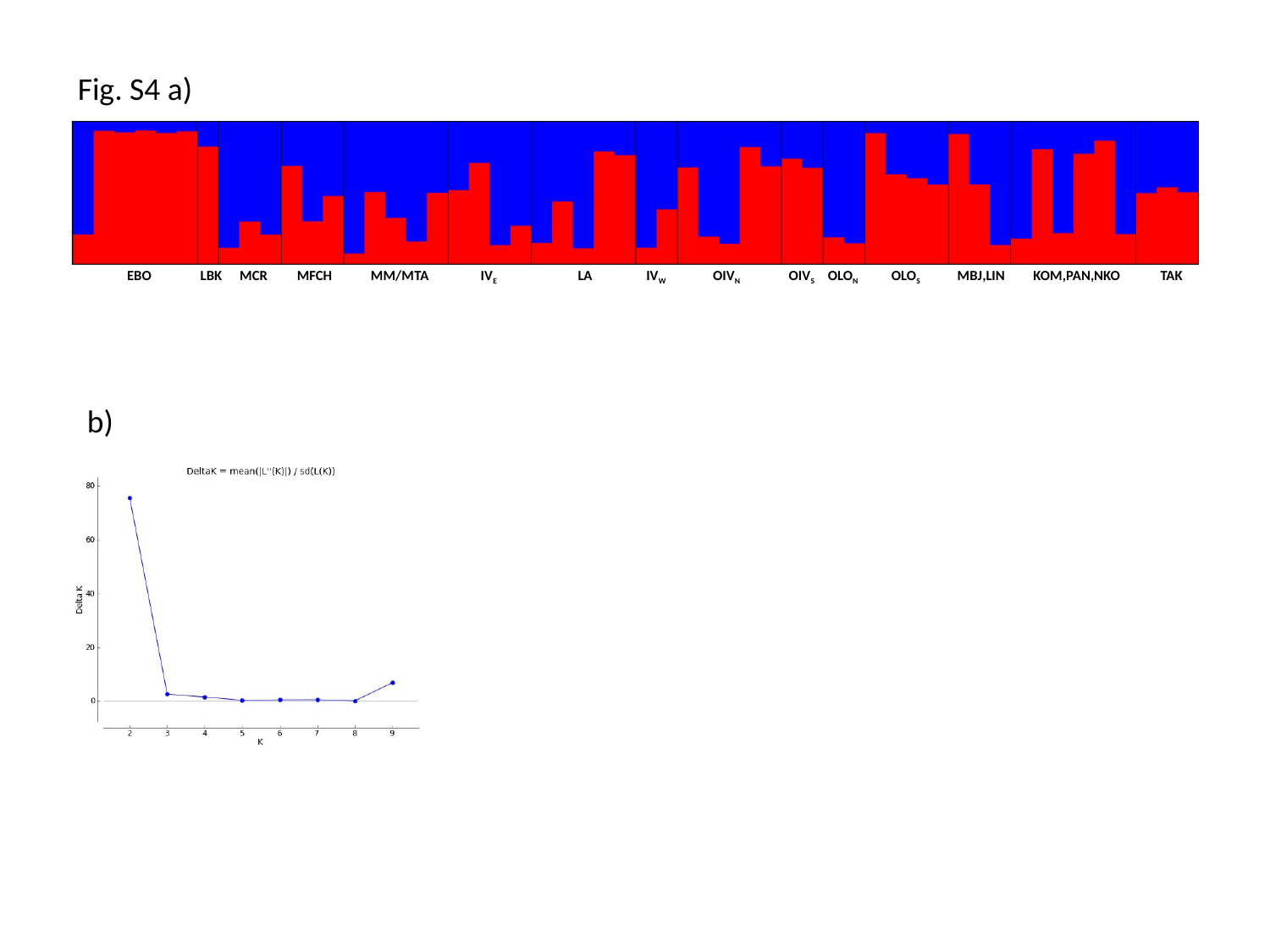

Fig. S4 a)
EBO
LBK
MCR
MFCH
MM/MTA
IVE
LA
IVW
OIVN
OIVS
OLON
OLOS
MBJ,LIN
KOM,PAN,NKO
TAK
b)

## Slide 7
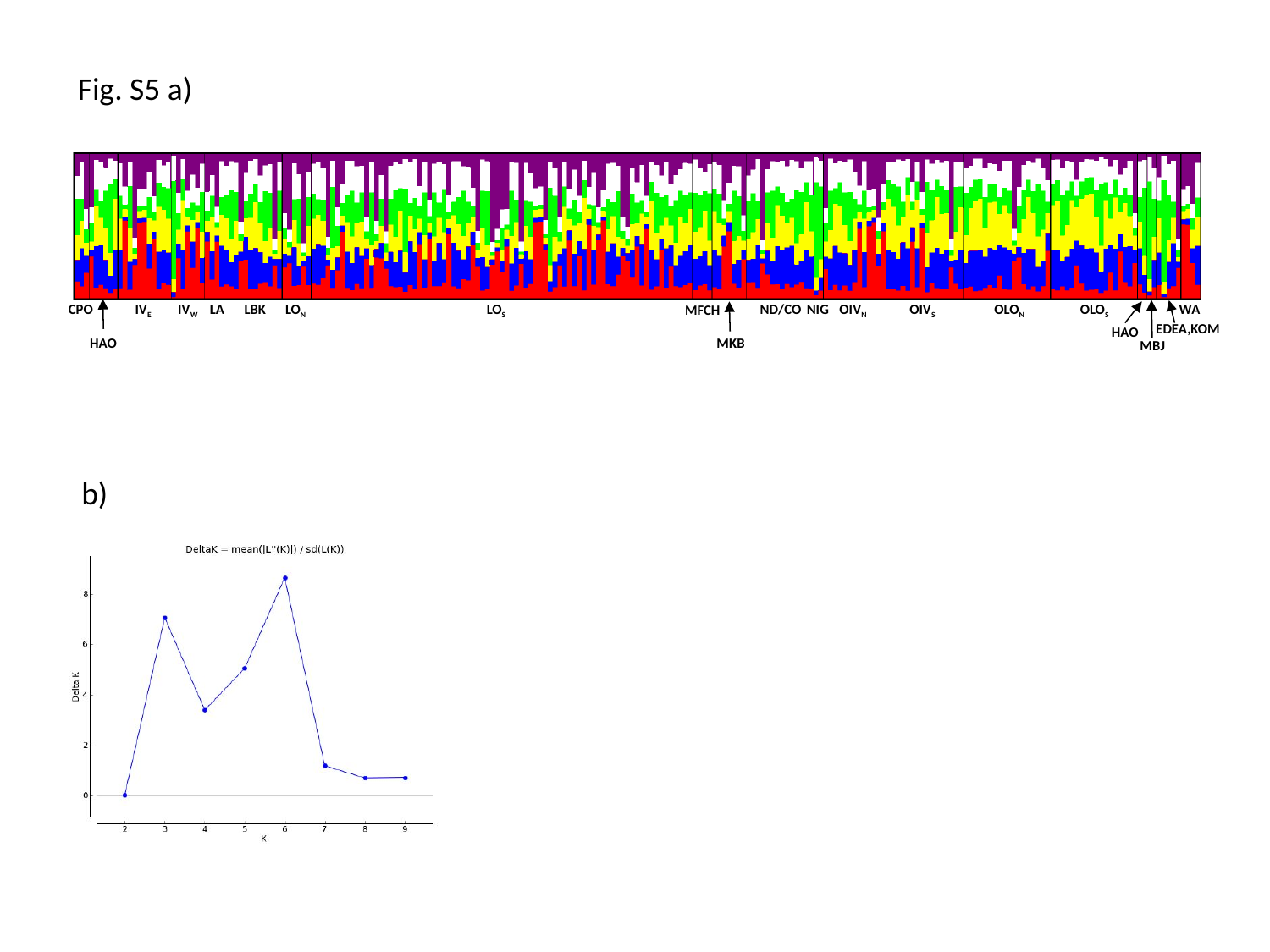

Fig. S5 a)
ND/CO
NIG
OIVN
OIVS
OLON
OLOS
WA
CPO
IVE
IVW
LA
LBK
LON
LOS
MFCH
EDEA,KOM
HAO
HAO
MKB
MBJ
b)

## Slide 8
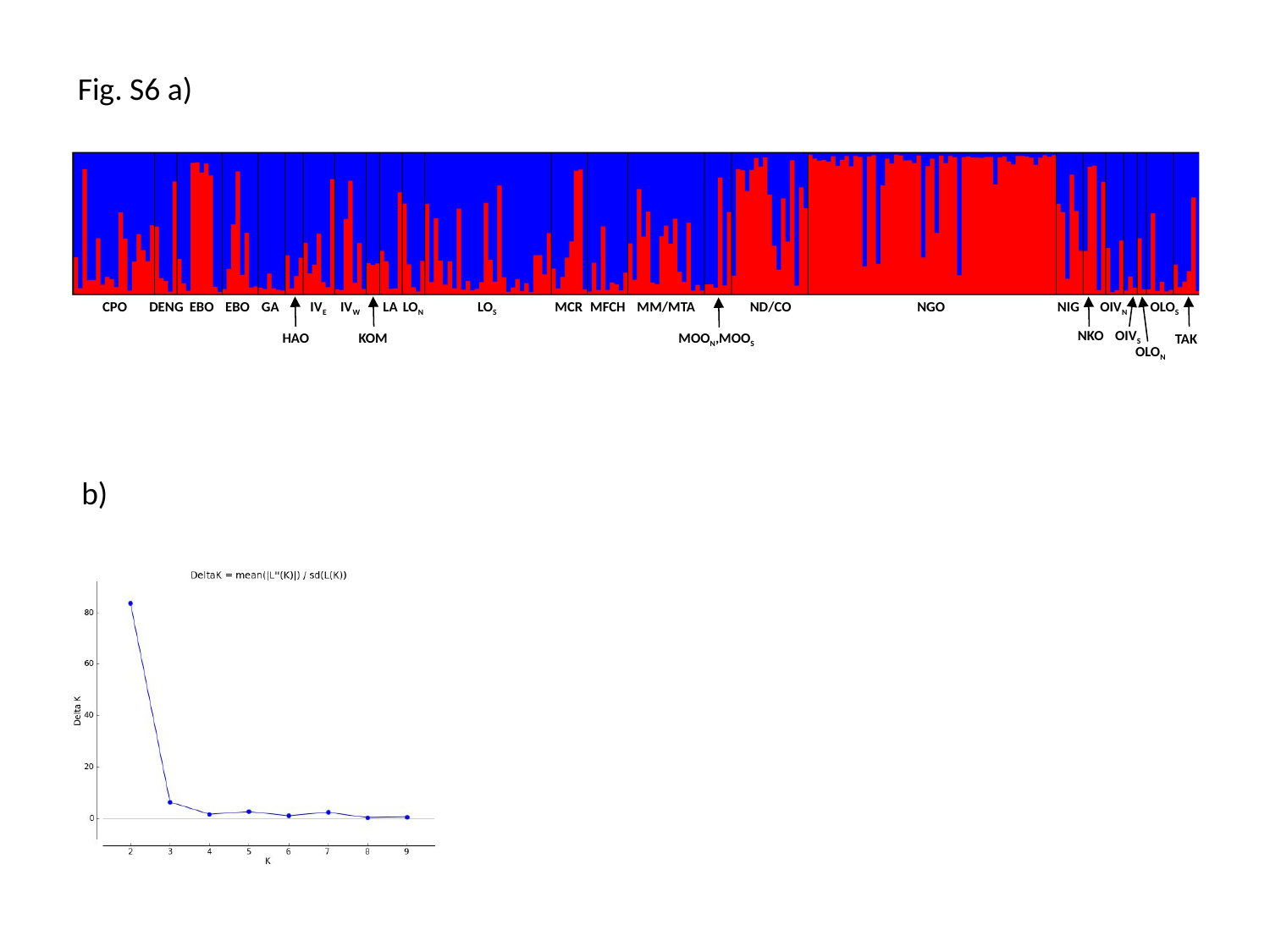

Fig. S6 a)
CPO
DENG
EBO
EBO
GA
IVE
IVW
LA
LON
LOS
MCR
MFCH
MM/MTA
ND/CO
NGO
NIG
OIVN
OLOS
OIVS
NKO
HAO
KOM
MOON,MOOS
TAK
OLON
b)

## Slide 9
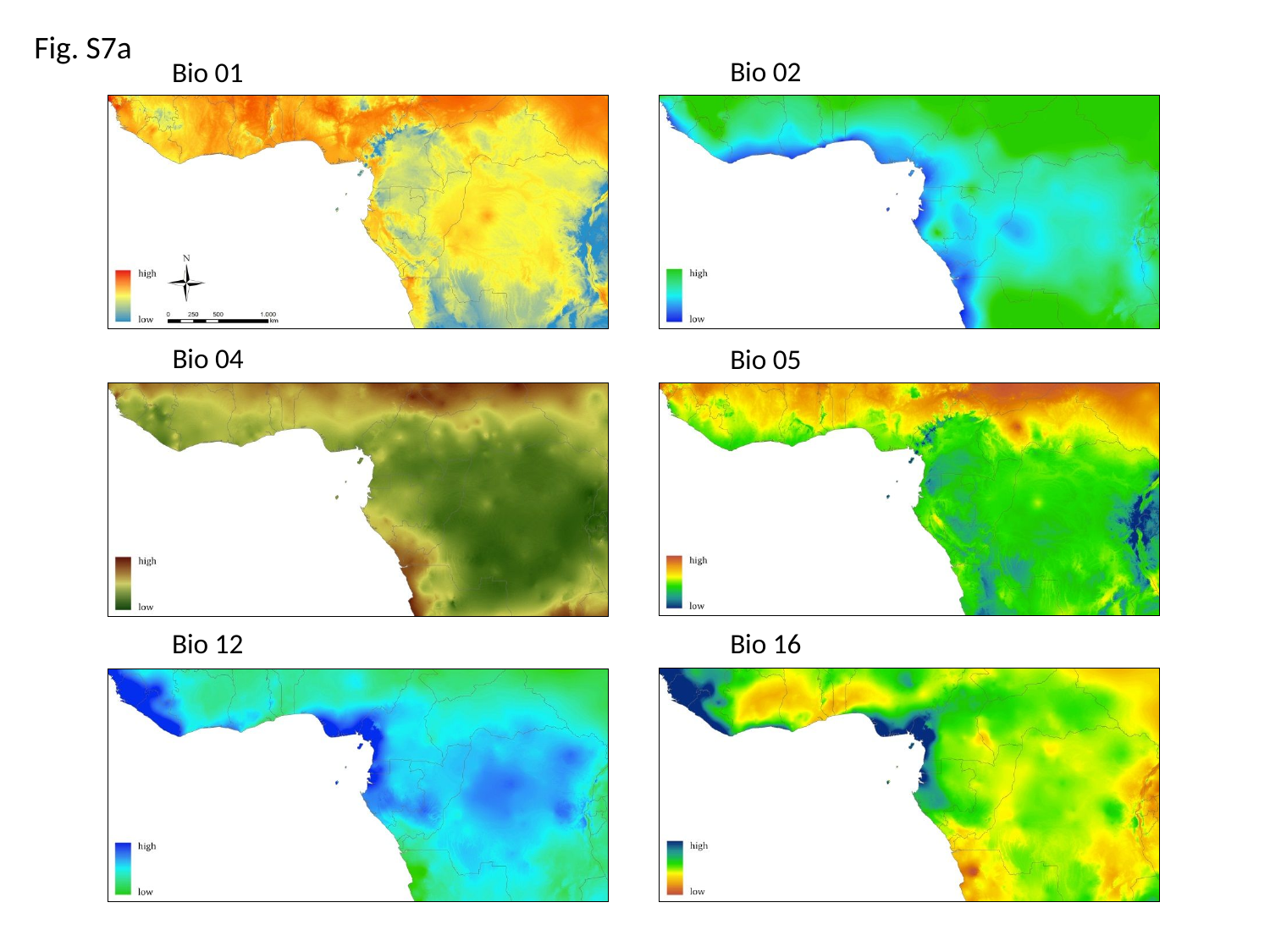

Fig. S7a
Bio 02
Bio 01
Bio 04
Bio 05
Bio 12
Bio 16

## Slide 10
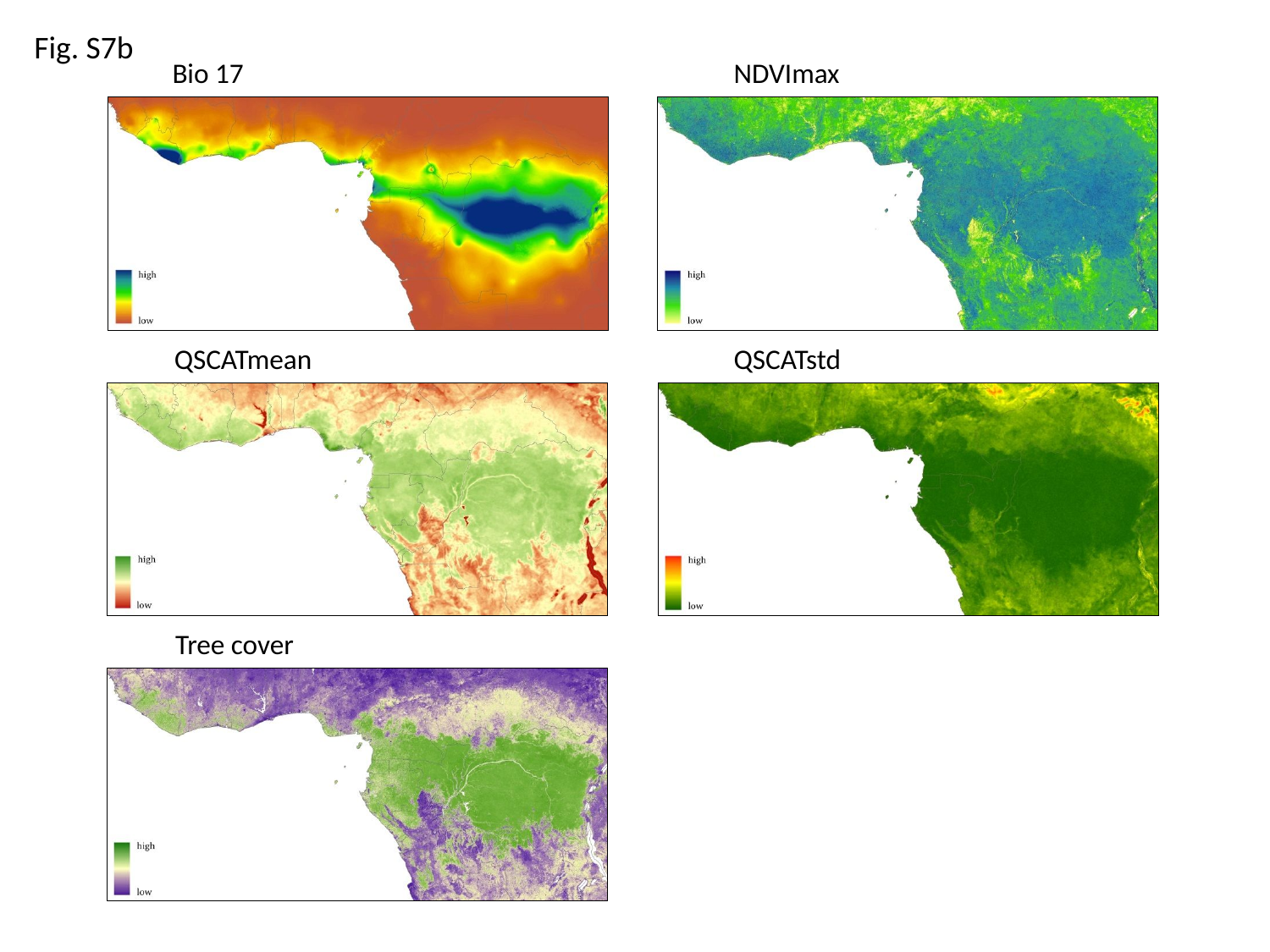

Fig. S7b
Bio 17
NDVImax
QSCATmean
QSCATstd
Tree cover

## Slide 11
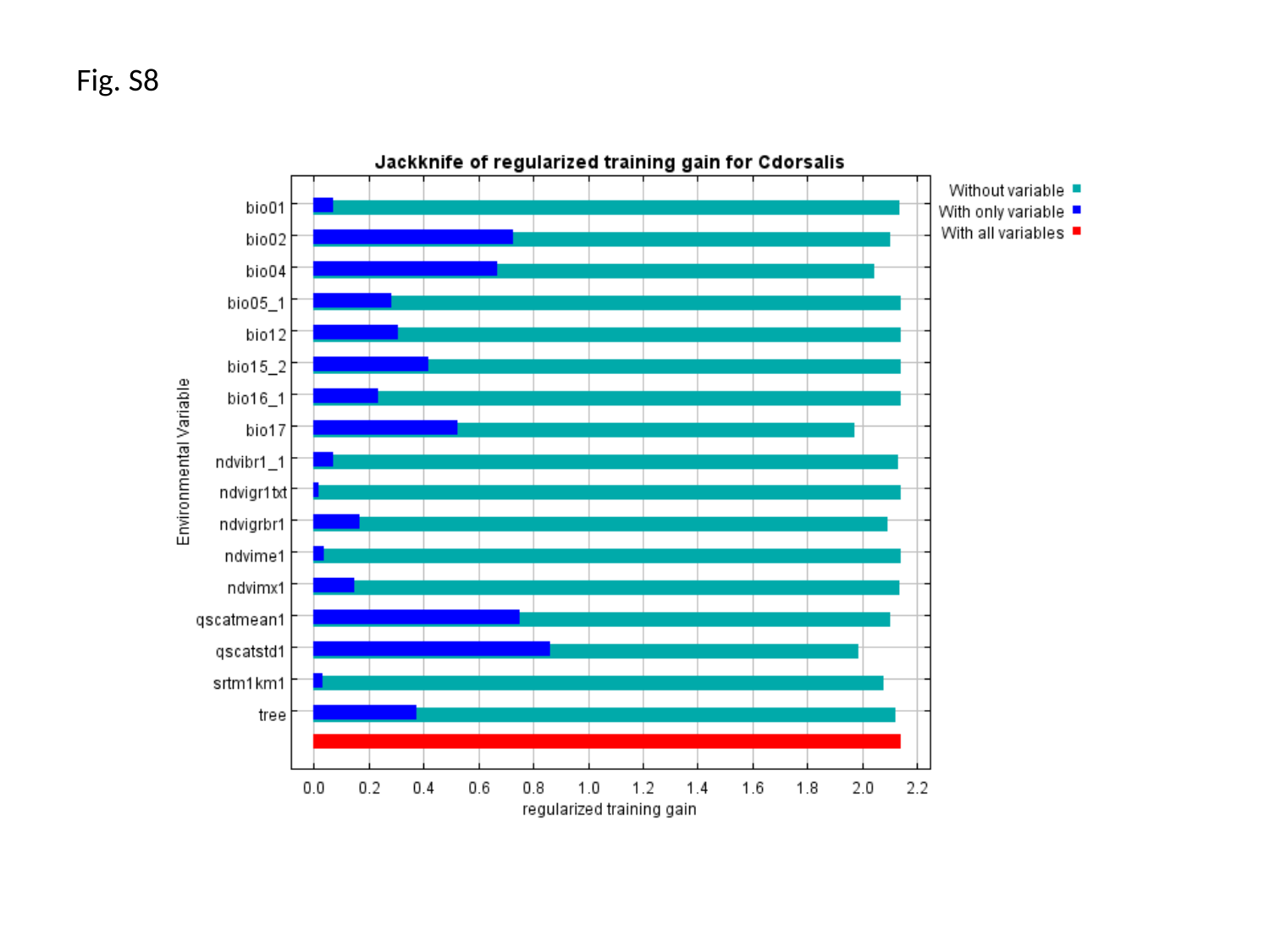

Fig. S8

## Slide 12
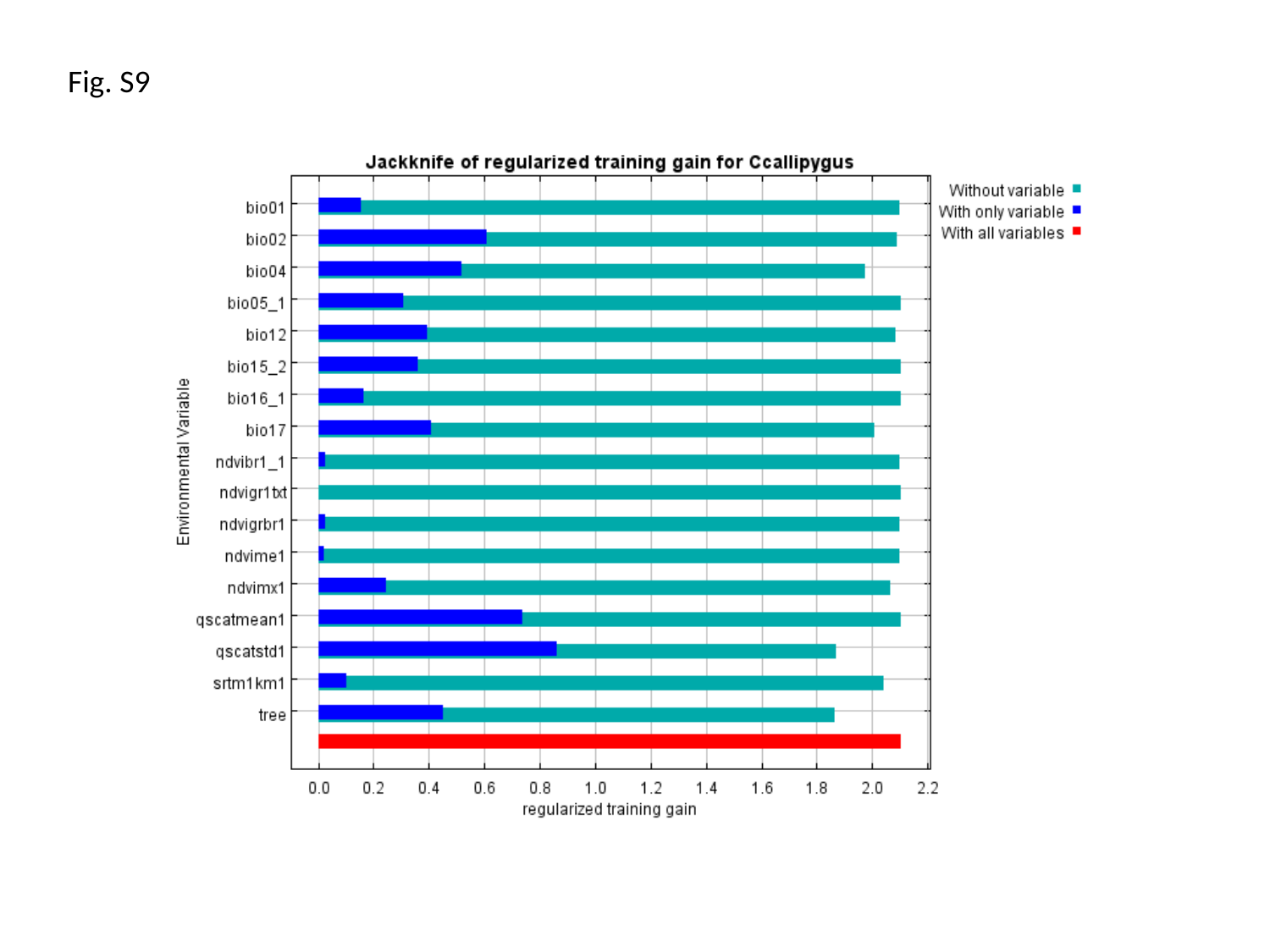

Fig. S9

## Slide 13
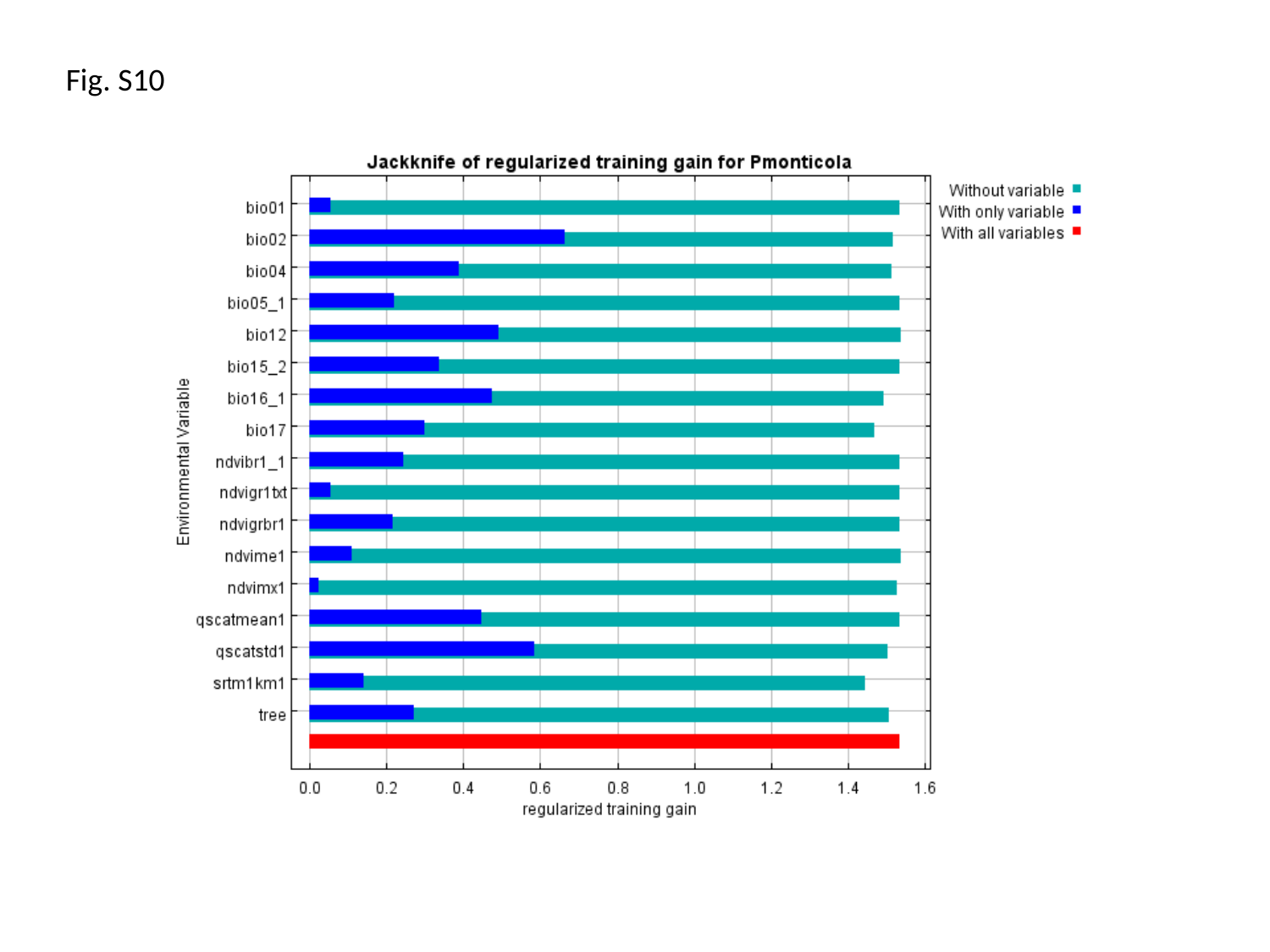

Fig. S10

## Slide 14
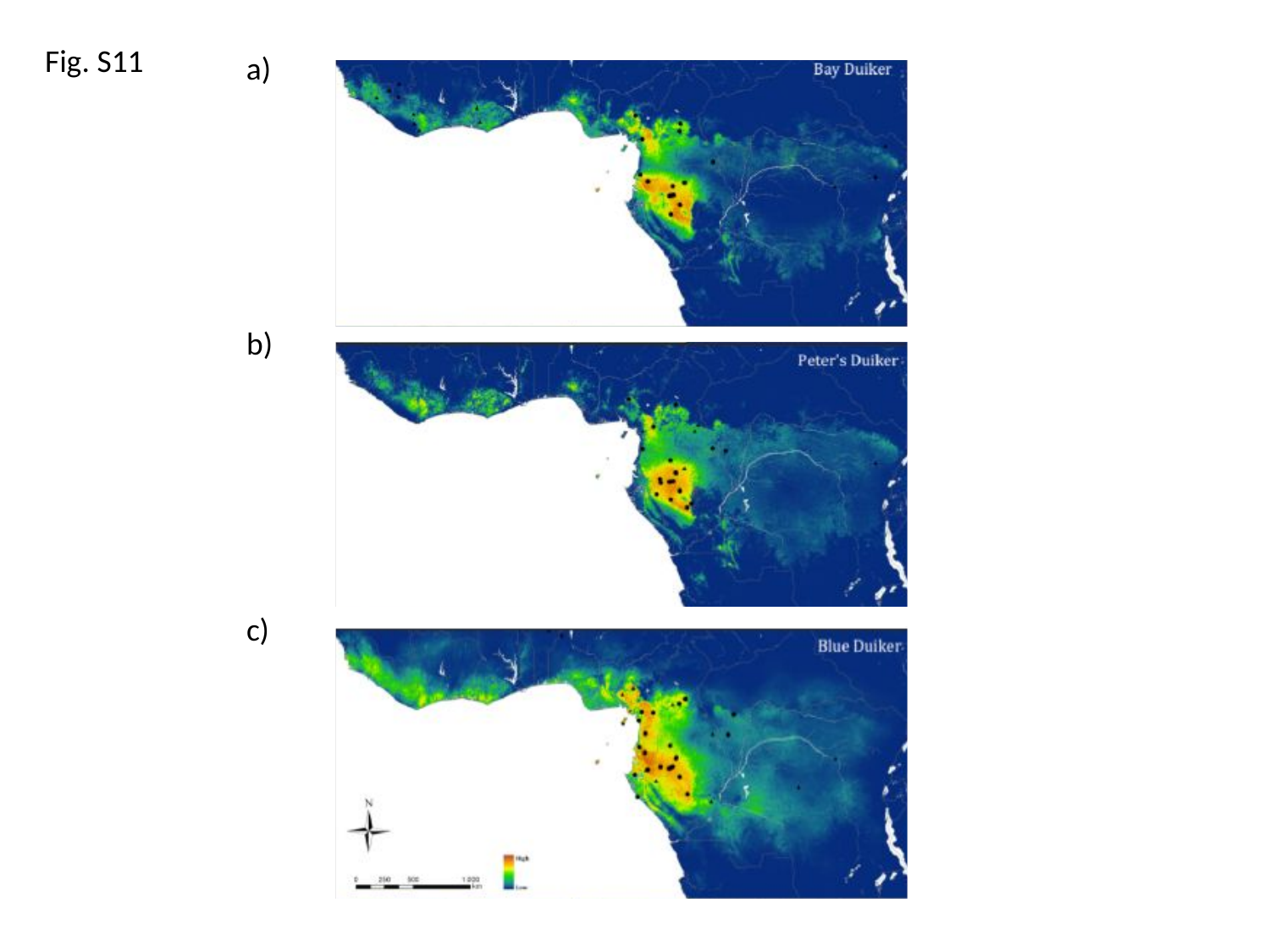

Fig. S11
a)
b)
c)

## Slide 15
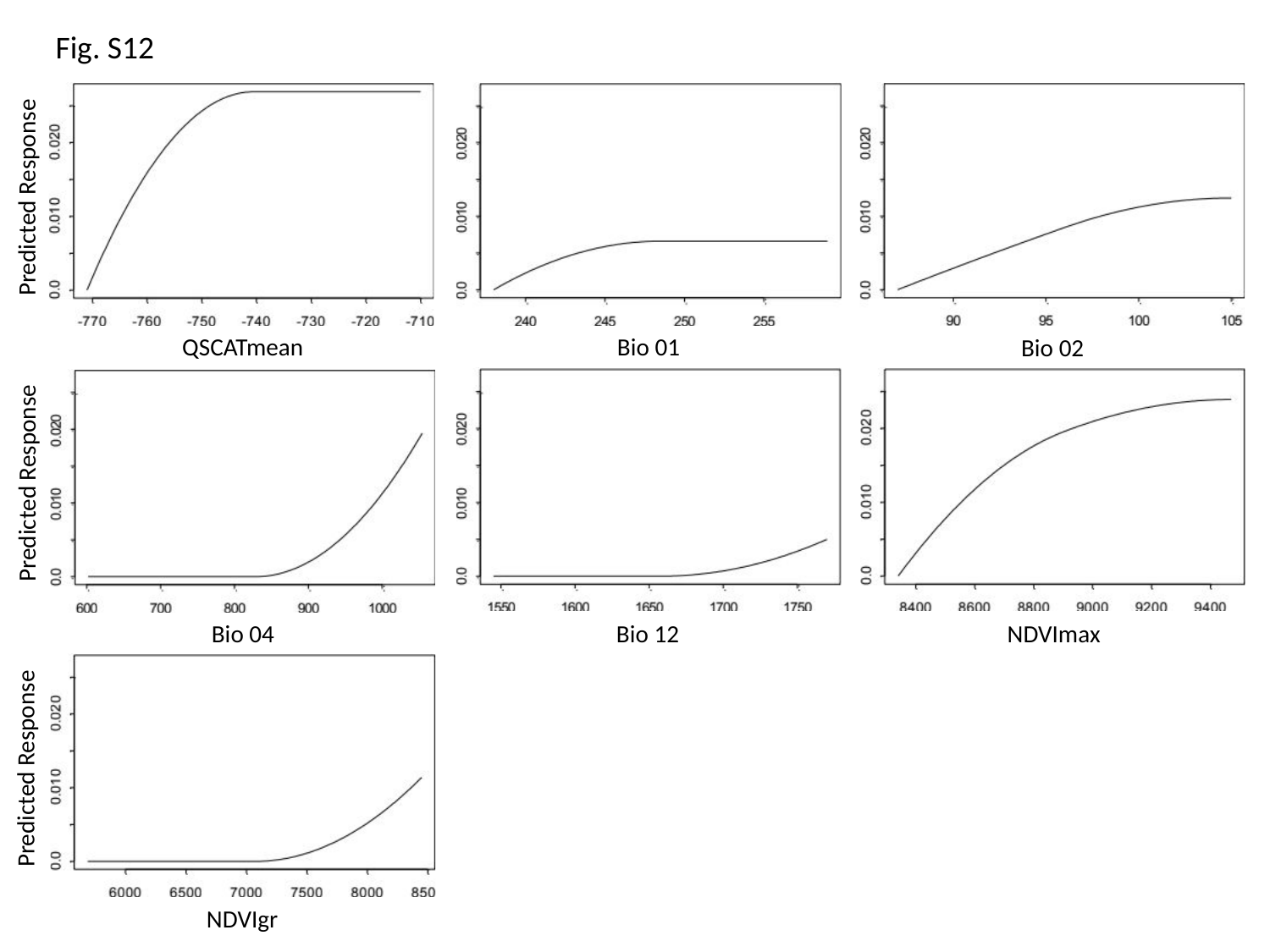

Fig. S12
Predicted Response
QSCATmean
Bio 01
Bio 02
Predicted Response
Bio 12
NDVImax
Bio 04
Predicted Response
NDVIgr

## Slide 16
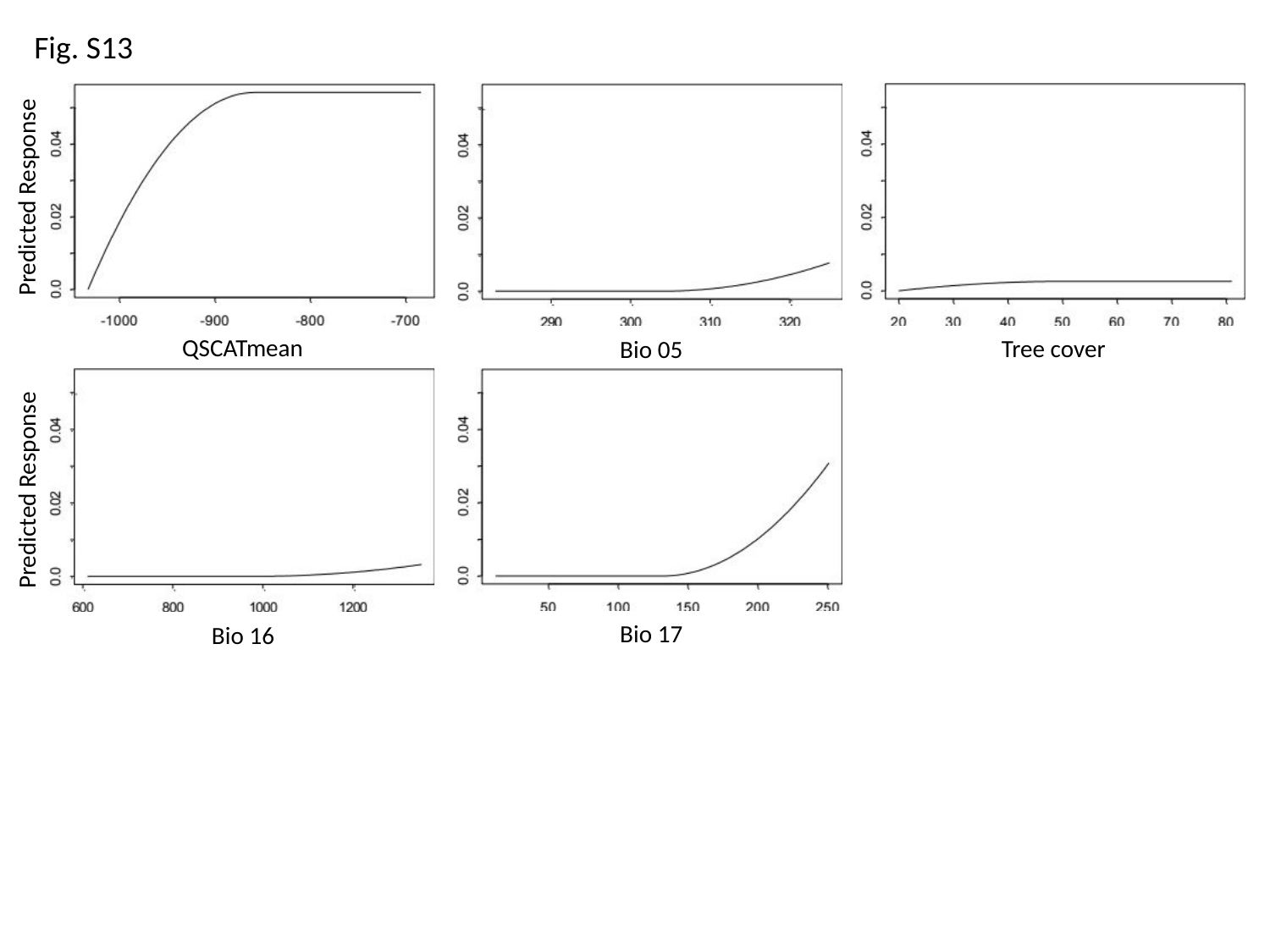

Fig. S13
Predicted Response
QSCATmean
Tree cover
Bio 05
Predicted Response
Bio 17
Bio 16
